# 15-deoxy-Δ^12,14^-Prostaglandin J_2_ inhibits human soluble epoxide hydrolase by a dual orthosteric and allosteric mechanism

**DOI:** 10.1038/s42003-019-0426-2

**Published:** 2019-05-17

**Authors:** Giancarlo Abis, Rebecca L. Charles, Jolanta Kopec, Wyatt W. Yue, R. Andrew Atkinson, Tam T. T. Bui, Steven Lynham, Simona Popova, Yin-Biao Sun, Franca Fraternali, Philip Eaton, Maria R. Conte

**Affiliations:** 1 Randall Centre for Cell and Molecular Biophysics, School of Basic and Medical Biosciences, King’s College London, London, SE1 1UL UK; 2 School of Cardiovascular Medicine & Science, The Rayne Institute, Lambeth Wing, St Thomas’ Hospital, King’s College London, London, SE1 7EH UK; 3 Structural Genomics Consortium, Nuffield Department of Medicine, University of Oxford, Oxford, OX3 7DQ UK; 4 Centre for Biomolecular Spectroscopy, King’s College London, London, SE1 1UL UK; 5 Proteomics Facility, Centre of Excellence for Mass Spectrometry, The James Black Centre, King’s College London, London, SE5 9NU UK

**Keywords:** Molecular biophysics, X-ray crystallography

## Abstract

Human soluble epoxide hydrolase (hsEH) is an enzyme responsible for the inactivation of bioactive epoxy fatty acids, and its inhibition is emerging as a promising therapeutical strategy to target hypertension, cardiovascular disease, pain and insulin sensitivity. Here, we uncover the molecular bases of hsEH inhibition mediated by the endogenous 15-deoxy-Δ^12,14^-Prostaglandin J_2_ (15d-PGJ_2_). Our data reveal a dual inhibitory mechanism, whereby hsEH can be inhibited by reversible docking of 15d-PGJ_2_ in the catalytic pocket, as well as by covalent locking of the same compound onto cysteine residues C423 and C522, remote to the active site. Biophysical characterisations allied with in silico investigations indicate that the covalent modification of the reactive cysteines may be part of a hitherto undiscovered allosteric regulatory mechanism of the enzyme. This study provides insights into the molecular modes of inhibition of hsEH epoxy-hydrolytic activity and paves the way for the development of new allosteric inhibitors.

## Abstract (web summary)

Giancarlo Abis et al. show that 15-deoxy-Δ12,14-Prostaglandin J2 inhibits the human soluble epoxide hydrolase by reversibly binding its catalytic pocket or by covalently binding reactive cysteines far from the active site. Inhibitors targeting these allosteric sites might prove to be useful therapeutic strategies.

## Introduction

Human soluble epoxide hydrolase (hsEH) is a cytosolic enzyme which modulates the metabolism of bioactive epoxy fatty acids (EpFAs), by converting them into their corresponding vicinal diols^1,2^. In mammals, the enzyme is composed of two structurally distinct domains connected by a proline-rich linker^3^. Whilst the N-terminal domain exerts phosphatase activity on glycerophospoholipids and lysophosphatidic acids^4–6^, the hydrolytic activity on EpFAs resides in the C-terminal domain (CTD)^7–9^. This hydrolysis involves a S_N_2 nucleophilic attack by D335 on the least hindered carbon atom of the epoxide, followed by water-mediated release of the alkyl-enzyme intermediate, assisted by D496 and H524. Residues Y383 and Y466 provide support to catalysis through the establishment of hydrogen bonds with the oxygen of the epoxy ring^1^. The catalytic site of hsEH CTD has been described as an ‘L-shaped’ tunnel with both ends open to the solvent. The catalytic triad (D335, D496 and H524) is located at the vertex of the ‘L’, edged by two hydrophobic regions at both sides, namely the F267 Pocket and W334 Niche^1,3^. The EpFA substrates of hsEH CTD are oxygenated derivatives of arachidonic, linoleic and α-linoleic acids, which possess anti-inflammatory, antihyperalgesic and vasoactive properties, and whose bioavailability has been linked to cardiovascular, inflammatory and neurodegenerative diseases, nociception and diabetes^10–12^. Of these substrates, the best characterised are the epoxyeicosatrienoic acids (EETs)^2,13,14^, which are endothelial-derived hyperpolarising factors able to induce dilation of small-size arteries in vascular districts of kidneys, heart, lung and brain^15–18^. EETs exhibit other beneficial properties, such as anti-platelet aggregation, anti-inflammatory effects, analgesic activity, apoptotic inhibition, vascular smooth muscle cell anti-migratory action and cardio-protection^16,19^. Given that hydrolysis of EETs to the corresponding diols, namely dihydroxyeicosatrienoic acids, leads to largely inactive compounds^13^, hsEH inhibition has emerged as a promising strategy to enhance the bioavailability of EETs and reap their protective and beneficial effects. The search for hsEH inhibitors has been the object of intense research for almost 30 years, leading to the discovery of more than a thousand 1,3-disubstituted urea antagonists, and related amides, carbamates, esters and carbonates, able to inhibit hsEH^20,21^. Of the numerous compounds developed, only few have reached clinical trials. The 12-(3-adamantan-1-yl-ureido)-dodecanoic acid (AUDA), and its salts and esters^22^, induced increased microvessel flux in healthy and heart failure human subjects, but its efficacy at a systemic level was not conclusively established^23^. The inhibitor AR9281 (1-adamantanyl-3-(1-acetylpiperidin-4-yl) urea), reduced systolic blood pressure in Angiotensin II-induced hypertensive mice, ameliorated renal injury recovery and improved vascular function^24^. The results of phase II clinical trials in patients with impaired glucose tolerance and mild to moderate hypertension were however inconclusive^25,26^. The compound GSK2256294 ((*1R,3S*)-(*cis*)-N-{[4-cyano-2-(trifluoromethyl)phenyl]methyl}−3-{[4-methyl-6-(methylamino)−1,3,5-triazin-2-yl]amino} cyclohexanecarboxamide) showed suppression of pulmonary inflammation in mice exposed to cigarette smoke^27^, though the results from phase II clinical trials with healthy volunteers, moderately obese smokers and mild to moderate hypertensive patients are still pending^28,29^. Given that no inhibitors characterised thus far has reached the clinic, research into new hsEH antagonists remains active.

At endogenous level, sEH activity was thought to be exerted largely by controlling protein expression level^17^. A breakthrough in the field was the discovery of a first endogenous ligand, namely 15-deoxy-Δ^12,14^-Prostaglandin-J_2_ (15d-PGJ_2_), able to efficiently modulate the activity of murine sEH^30^. 15d-PGJ_2_ is a product of the arachidonic acid metabolism, generated in vitro by macrophages in response to inflammatory stimuli, and identified in vivo in atherosclerotic plaques^31^, as well as in plasma during the resolution phase of inflammation^32,33^. As an *α/β*-unsaturated ketone electrophilic lipid, 15d-PGJ_2_ reacts in Michael additions with cysteine residues of proteins^34^, including Kelch-like ECH-associated protein 1 (Keap1)^35^, negative regulator of the nuclear factor (erythroid-derived-2)-like 2 (Nrf2)^36^, peroxisome proliferator-activated receptor gamma (PPARγ)^35,37^ and IκB kinase (IKK)^38,39^, modulating their function and/or cellular localisation. Charles et al.^30^ showed that 15d-PGJ_2_ induces vasodilation of coronary vasculature of *ex vivo* perfused murine hearts in a sEH-dependent manner. Given that a knock-in C521S-sEH murine model showed resistance to 15d-PGJ_2_-mediated vasodilation, the cysteine residue C521 (C522 in human sequence numbering) was identified as the target of the Michael addition by the electrophilic lipid. The molecular details of this inhibition though remained unclear.

By using a combined biochemical and biophysical approach, this study elucidates the mechanism of human sEH inhibition by 15d-PGJ_2_. hsEH was found to be covalently modified by 15d-PGJ_2_ on two cysteine residues located outside the catalytic pocket, one of which, C423, was to our knowledge discovered here for the first time, as it is not present in the murine ortholog. Most importantly, we revealed that the covalent modification of both cysteines is accompanied by a conformational change of the protein, thereby uncovering a hitherto unknown allosteric mechanism of sEH inhibition. In addition to the allosteric control, our investigations show that 15d-PGJ_2_ is also able to inhibit hsEH orthosterically, by interacting in a reversible non-covalent manner with residues within the catalytic pocket. We therefore propose a dual molecular model of 15d-PGJ_2_-mediated hsEH inhibition, whereby the ligand can bind reversibly to hsEH impeding the catalysis or adduct covalently the enzyme on allosteric sites causing a conformational switch towards an inactive state.

## Results

### 15d-PGJ_2_ covalently modifies two cysteines in hsEH CTD

**Fig. 1:**
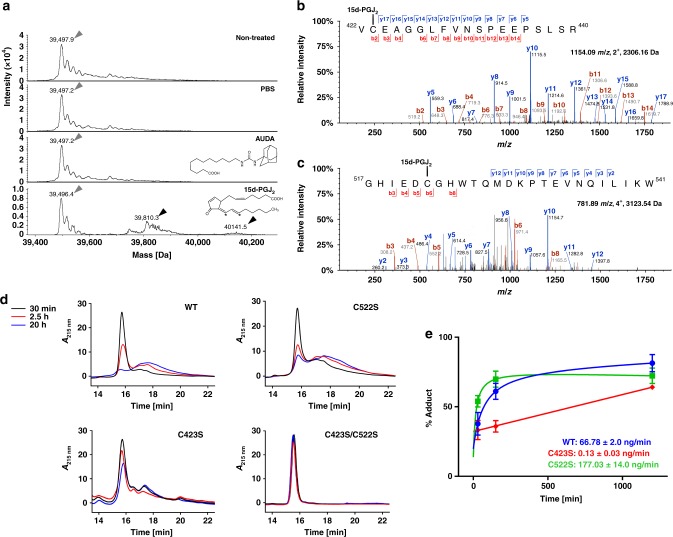
Analysis of the covalent interaction between hsEH CTD and 15d-PGJ_2_. **a** ESI-MS experiments. Grey and black arrows indicate the free and covalently modified hsEH CTD, respectively. The electrophilic carbon atoms of 15d-PGJ_2_ are indicated by asterisks. **b** LC-MS/MS evidence of C423 modification. A peptide with *m/z* 1154.09^2+^ was assigned through the identification of ions b2-b4, b6-b14 and y5-y17. The direct assignment of the modification on both b2-ions and y17-ions was strong evidence of modification of C423. **c** LC-MS/MS evidence of C522 modification. The peptide exhibited a *m/z* of 781.89^4+^. Its sequence was assigned through the detection of b3-b6, b8 and y2-y12 ions. The b6 ion modification allowed the direct identification of C522 adduction. **d** HPLC separation of the covalent complexes. The traces were collected at three different time points, upon incubation of hsEH CTD and 15d-PGJ_2_. For clarity, the chromatograms of the free proteins are reported in Supplementary Fig. 1. **e** 15d-PGJ_2_ rate of adduction. Rates were calculated from deconvolution of the HLPC traces shown in **d**, as described in the Methods section. The mutant C423S showed a considerably lower rate of adduction to C522, suggesting that the kinetics of modification of the two residues are different. Data presented as average ± SEM of *n* = 4 WT, *n* = 3 C522, and *n* = 3 C423S. (Source data available in Supplementary Data 1)

To investigate whether human sEH C-terminal Domain (hsEH CTD) was covalently modified by the endogenous electrophilic lipid 15d-PGJ_2_, as reported for the murine ortholog^30^, electrospray ionisation mass spectrometry (ESI-MS) experiments were performed. Upon incubation of the human protein with the prostaglandin, three main peaks were detected (Fig. 1a): whilst one matched the free protein molecular mass (39496.4 Da), the other two showed a deconvoluted mass of 39810.3 and 40141.5 Da, corresponding to the addition respectively of 312.1 and 645.1 Da. These peaks were assigned to the covalent complexes formed between hsEH CTD and 15d-PGJ_2_ molecules (316.4 Da), revealing that the protein is modified in vitro by either one or two units of prostaglandin. No signal other than the apoprotein was observed upon treatment with the reversible antagonist AUDA or buffer alone. Liquid chromatography-tandem mass spectrometry (LC-MS/MS) revealed two distinct sites of adduction for 15d-PGJ_2_: C522 (Fig. 1b), located at the entrance of the F267 Pocket, which corresponds to the murine counterpart C521 previously identified;^30^ and C423 (Fig. 1c), a residue conserved only in Primates (see below), located outside the active site, approximately 10 Å away from the edge of the F267 Pocket.

We next asked whether these two modification sites on the human sEH protein were equivalent. To evaluate the kinetics of adduction on the two cysteines, we followed the appearance overtime of the covalent adducts, comparing the behaviour of wild-type hsEH CTD (WT) with that of single C423S and C522S mutants, and C423S/C522S double mutant, replacing the reactive cysteines with serines. The proteins incubated with 15d-PGJ_2_ were analysed via HPLC reverse phase chromatography at various time points (Fig. 1d, Supplementary Fig. 1). The UV elution profiles for wild type (WT), C423S and C522S proteins showed the presence of additional species that were retained in the reverse phase column longer than apoproteins, which were assigned to the covalently modified adducts. The lack of additional elution peaks for the C423S/C522S mutant corroborated the identity of the retained species and confirmed that C423S and C522S are the only targets of the Michael addition of 15d-PGJ_2_ to hsEH CTD. Deconvolution of HPLC UV traces provided a measure for the rate of adduct formation overtime, showing that the covalent modification of C423S and C522S occurs at different rates. Interestingly, the C423S mutant experiences a considerably lower adduction rate, indicating that C423 reacts with 15d-PGJ_2_ faster than C522 (Fig. 1e).

### 15d-PGJ_2_ covalent adduction on C423 and C522 inhibits hsEH

**Fig. 2:**
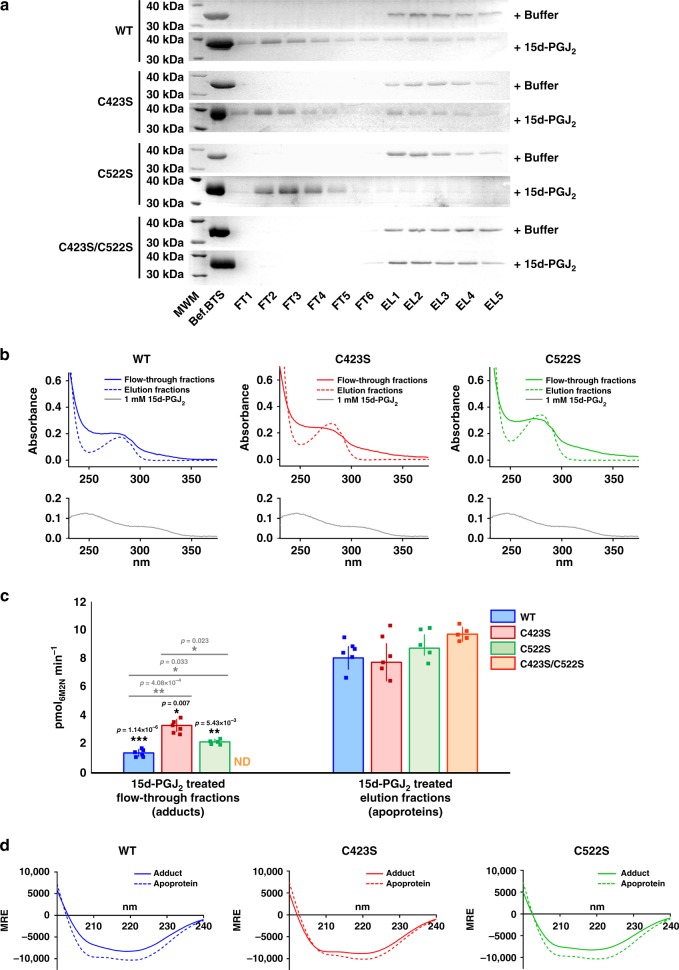
Analyses of 15d-PGJ_2_-hsEH CTD covalent complexes. **a** Purification of 15d-PGJ_2_-hsEH CTD covalent adducts. SDS-PAGE analysis of the BTS purification of the proteins when incubated with either buffer (control) or 15d-PGJ_2_ (MWM, molecular weight marker; FT, flow-through fraction; EL, elution fraction). All the proteins bound to the BTS resin in the control treatment with buffer alone. Upon treatment with 15d-PGJ_2_, a fraction of the WT, C423S and C522S enzymes was not retained by the resin and was collected in the flow-through. The C423S/C522S mutant interacted with the BTS in all conditions. Full uncropped gels are reported in Supplementary Fig. 2. **b** UV analyses of the covalent adducts. The figure reports the UV spectra of the BTS fractions collected either in the flow-through or in the elution fractions. The peaks at 250 and 330 nm confirmed 15d-PGJ_2_ adduction to the proteins collected in the BTS flow-through (from panel **a**). **c** Comparative enzymatic activity analysis. The enzymatic activity of hsEH covalent adducts and apoproteins were measured with a spectrofluorimetric assays. The comparison showed a significant reduction in hydrolytic activity for the covalent complexes (ND, non-determined; as the C423S/C522S did not generate any adduct). The asterisks refer to one-tailed homoscedastic *t*-test (apoprotein vs. adducts), and the significance is indicated as follows: *0.05 < *p* ≤ 0.005, **0.005 < *p* ≤ 0.0005, ***p < 0.0005. Data presented as average ± SEM of *n* = 6 WT, *n* = 6 C522, *n* = 5 C423S, and *n* = 5 C423S/C522S. (Source data available in Supplementary Data 2). **d** CD analyses. Comparison of the CD spectra of the apoproteins (WT, C423S and C522S) and the corresponding 15d-PGJ_2_ covalent adducts revealed a conformational rearrangement upon 15d-PGJ_2_ modification (see Table 1). Spectroscopic and enzymatic analyses of the other fractions collected in the purifications are reported in the Supplementary Fig. 3

We interrogated the effect of Michael addition of 15d-PGJ_2_ to C423 and C522 on hsEH CTD enzymatic activity. To purify the covalent adducts, a bespoke separation strategy via Benzylthio-Sepharose (BTS) affinity chromatography resin was devised. BTS is able to bind sEH through interactions between the enzyme’s hydrophobic active site and benzylmercaptan moieties immobilised onto Sepharose beads^40,41^. We hypothesised that the modification by 15d-PGJ_2_ might affect this interaction, possibly through conformational effects (see below). Upon incubation with 15d-PGJ_2_, a proportion of the WT, C423S and C522S enzymes was indeed not retained by the BTS resin (Fig. 2a and Supplementary Fig. 2). These flow-through fractions were unambiguously shown to contain prostaglandin-covalently adducted proteins that failed to bind to the resin (Fig. 2b): their UV spectra in fact revealed a three-peak absorbance profile, with the expected aromatic amino acids absorbance at 280 nm and two additional peaks at 250 and 330 nm, which matched the UV spectrum of 15d-PGJ_2_ (Fig. 2b, Supplementary Fig. 3a). Not only did this indicate a successful separation of prostaglandin-modified proteins from the apoproteins, but also suggested that in the covalently adducted species the active site of the enzyme is somewhat altered, becoming unable to bind to the benzylmercaptan compound.

The epoxy-hydrolytic activity of the purified hsEH proteins covalently modified by 15d-PGJ_2_ was then evaluated using a fluorimetric-based assay^42^ (Fig. 2c, Supplementary Fig. 3b). These experiments showed that the hsEH-15d-PGJ_2_ adducts experienced a reduction of enzymatic activity when compared to apoproteins, with the single modification of C423S and C522S mutants inhibiting the enzyme to a lesser extent. This suggests that the two cysteine residues are not equivalent in their ability to impair enzyme activity when covalently modified, and that their inhibitory effect is somewhat cumulative.

Although the covalent modification on C522 and C423 inhibits hsEH CTD, these cysteines are not located within the enzyme’s catalytic pocket, nor are they involved in the mechanism of epoxide hydrolysis. With C522 proximal to the entrance of the catalytic tunnel on the F267 pocket, the covalently bound 15d-PGJ_2_ moiety at this site may block access of the substrate to the active pocket through steric hindrance. Nonetheless, this is not a plausible explanation for C423, which is further away from the catalytic crevice. To understand in greater depth the molecular bases of this inhibitory mechanism, and prompted by the behaviour of the 15d-PGJ_2_ adducts failing to bind the BTS column, we next performed circular dichroism (CD) analysis (Fig. 2d, Supplementary Fig. 3c). Far-UV CD profiles of WT-15d-PGJ_2_, C423S-15d-PGJ_2_ and C522S-15d-PGJ_2_ covalent adducts differed from the spectra of the respective apoproteins in overall shape and molar ellipticities, thus reflecting changes in protein secondary structure content upon covalent binding. Deconvolution analyses of CD data indicate that thiol modification by 15d-PGJ_2_ is accompanied by a decrease in α-helical and an increase of β-strand content, and that double adduction of both cysteine residues results in a somewhat cumulative effect (Table 1). This indicates that the Michael addition of 15d-PGJ_2_ to both C423 and C522 induces conformational changes in the enzyme that could account for the impairment of hydrolytic activity. In other words, an allosteric mechanism of inhibition^43^.

**Table 1:** CD data deconvolution. Secondary structure content analyses indicate a decrease in α-helical and an increase in β-strand content upon modification

	α-helices (%)	β-strands (%)	Turns (%)	Unordered (%)
*WT*				
Adducts	22.5	35.5	21.9	29.6
Apoprotein	33.4	17.3	22.0	29.5
*C423S*				
Adduct	22.5	24.4	22.7	27.3
Apoprotein	29.2	18.2	22.4	30.6
*C522S*				
Adduct	26.5	19.6	23.0	29.1
Apoprotein	32.0	17.5	21.2	29.3

### 15d-PGJ_2_ reversibly binds the catalytic site of hsEH CTD

**Fig. 3:**
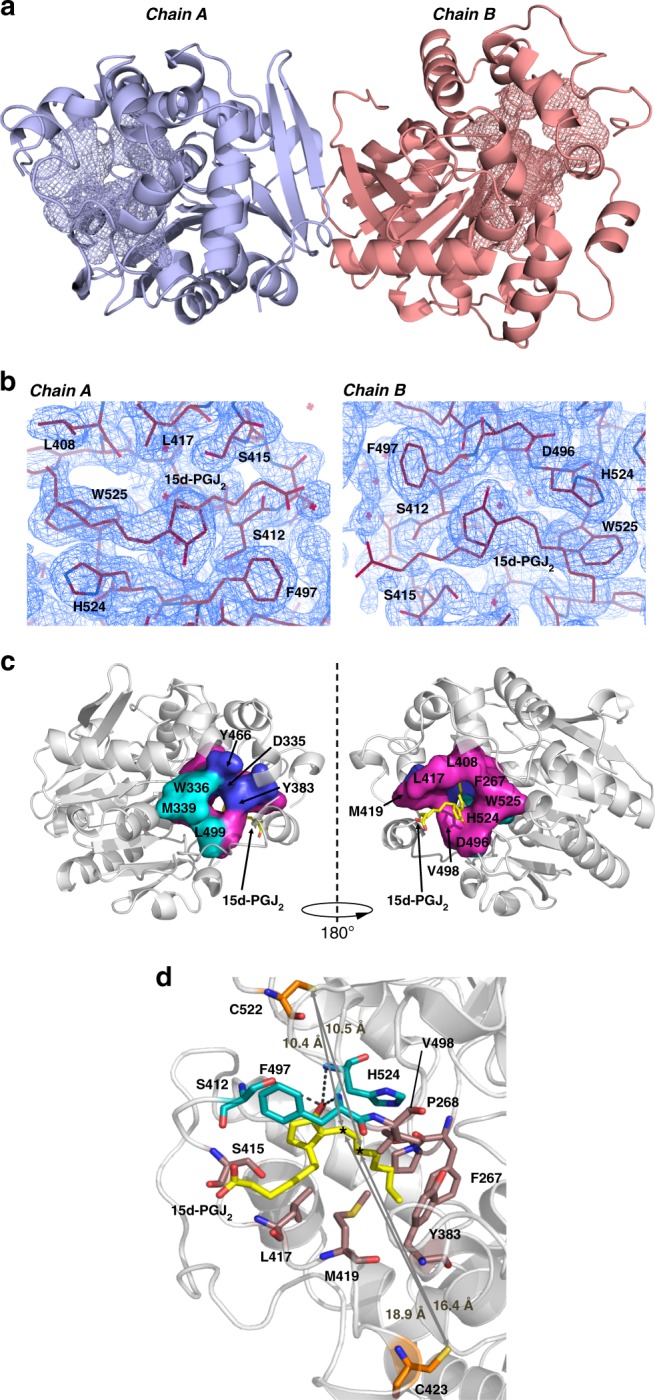
X-ray crystallographic structure of the 15d-PGJ_2_-hsEH CTD complex. **a** Crystallographic symmetry. The crystallographic homodimer is represented as a cartoon model (chain A and B in light blue and salmon red respectively), with the ‘L’-shaped catalytic site in mesh. **b** Electron density of the 15d-PGJ_2_ bound to hsEH CTD. The 2Fc-Fo map was contoured at 1 sigma. **c** 15d-PGJ_2_ binding site. The ‘L-shaped’ catalytic site of hsEH CTD is represented with a three-colours code: the F267 Pocket in magenta; the W336 Niche in cyan; the ‘L’ vertex formed by the catalytic D335 and the epoxide positioners Y363 and Y466 are represented in blue. **d** Overall 15d-PGJ_2_ interaction with hsEH CTD. The amino acids involved in van der Waals interaction are depicted in brown, while those forming hydrogen bonds with 15d-PGJ_2_ are in dark cyan. C423 and C522 are highlighted in orange, and the reactive electrophilic C13 and C15 atoms of the ligand are indicated by asterisks

To elucidate the basis of the 15d-PGJ_2_-mediated inhibitory mechanism, we pursued a structural approach. First, we solved the structure of apo hsEH CTD by X-ray crystallography, and our results were found to be in excellent agreement with previously deposited structures of the full-length enzyme^9^, with an RMSD of 0.257 Å on all CTD heavy atoms. The apoprotein crystallised in space group I2, with two hsEH CTD molecules in the asymmetric unit forming a homodimer (Fig. 3a, Supplementary Fig. 4; Table 2). To obtain the structure of the covalent adducts, hsEH CTD crystals were subjected to soaking with 15d-PGJ_2_ (Table 2). Although one unit of the prostaglandin was found bound to each monomer of hsEH CTD in the crystals, it was not covalently attached to either C522 or C432, but instead was accommodated in the F267 Pocket of the catalytic site (Fig. 3b, c). This structure therefore depicts an orthosteric non-covalent interaction between 15d-PGJ_2_ and hsEH CTD. Superposition of the apo and holo crystal structures gave an RMSD on all heavy atoms of 0.198 Å, indicating a high similarity of the two structures. Minor changes were observed in two regions proximal to the F267 Pocket (Supplementary Fig. 5).

**Table 2:** Data collection statistics for hsEH CTD in apo-state and complexed to 15d-PGJ_2_

	Apo hsEH CTD	Complexed hsEH CTD
*Data collection*		
Space group	I 1 2 1	I 1 2 1
*Cell dimensions*		
*a*, *b*, *c* (Å)	88.22, 80.14, 104.70	89.49, 79.99, 104.54
α, β, γ (°)	90.00, 95.39, 90.00	90.00, 96.78, 90.00
Resolution (Å)	29.60–2.60 (2.72–2.60)	29.73–2.00 (2.05–2.00)
*R*_sym_ (%)	9.1 (66.4)	9.6 (63.5)
*I*/σ*I*	5.7 (2.1)	10.2 (2.3)
Completeness (%)	99.9 (100.0)	99.2 (91.6)
Redundancy	5.1 (5.0)	5.0 (4.6)
*Refinement*		
Resolution (Å)	2.60	2.0
No. reflections (observed/unique)	114,202/22,492	244,141/48,933
*R*_work_/*R*_free_ (%)	24.6/27.7	17.4/21.9
*No. atoms*		
Protein	5086	5103
Ligand/ion	N/A	46
Water	13	226
*B*-*factors (Å*^*2*^*)*		
Protein	45.5	34.0
Ligand/ion	N/A	65.3
*R.m.s. deviations*		
Bond lengths (Å)	0.0104	0.0168
Bond angles (°)	1.4487	2.0545

In the reversible non-covalent complex with 15d-PGJ_2_, the di-unsaturated aliphatic chain and the ring of the ligand threaded inside the F267 pocket, whilst the mono-unsaturated aliphatic chain was found partially protruding outside the catalytic site (Fig. 3d). The di-unsaturated aliphatic chain established van der Waals contacts with protein residues of a hydrophobic surface formed by F267, P268, Y383, L408, L417 and V498. The oxygen of the 15d-PGJ_2_ ring engaged in a hydrogen bond with the backbone nitrogen of F497. F497 also made van der Waals contacts with the ligand benzylic moiety. Moreover, the hydroxyl group of S412 and the backbone nitrogen of H524 were found at a relatively short distance (around 3 Å) from the cyclopentenonyl oxygen of 15d-PGJ_2_, suggesting that they may participate with F497 in positioning the prostaglandin ring. Further van der Waals contacts were established between Cβ and Cγ of H524 and 15d-PGJ_2_ di-unsaturated aliphatic chain, S412 and the cyclopentenonyl ring of the prostaglandin, and S415, L417 and M419 and the mono-unsaturated aliphatic chain of the ligand.

**Fig. 4:**
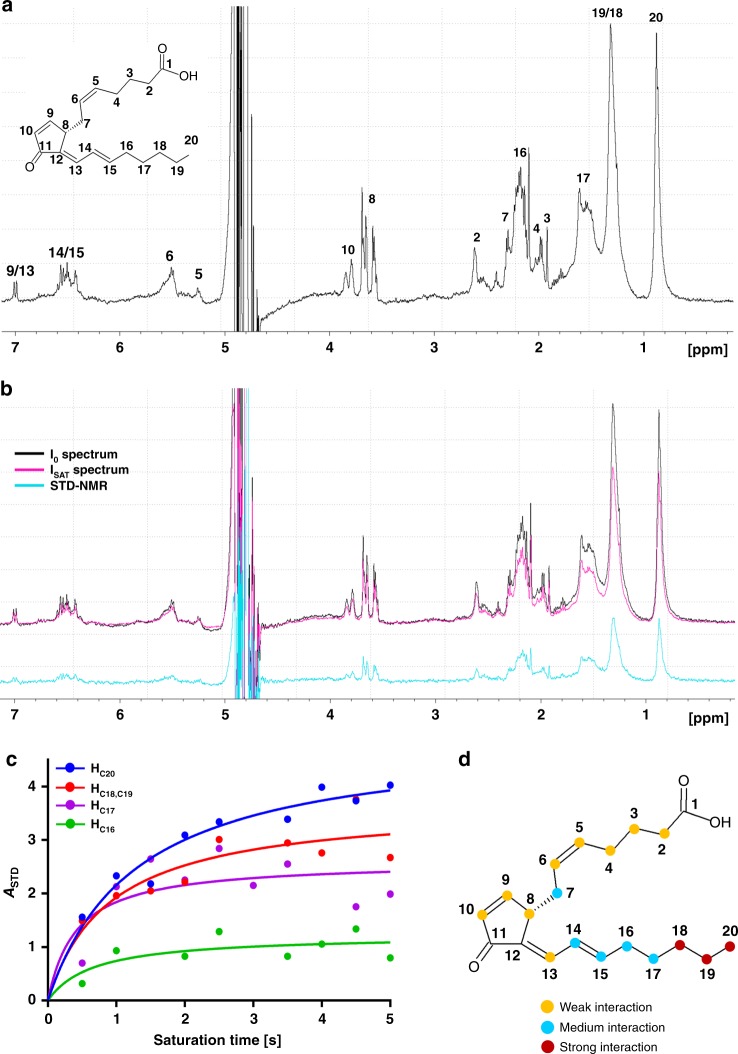
Saturation-transfer difference nuclear magnetic resonance (STD-NMR) experiments. **a** ^1^H-NMR spectrum of 1 mM 15d-PGJ_2_ and resonance assignment. **b** Comparison between *I*_0_ (black) and *I*_SAT_ (magenta) spectra. The difference spectrum (cyan) upon 6 s irradiation confirmed the reversible interaction between hsEH CTD and 15d-PGJ_2_ in solution. STD values were not corrected by T1. **c** STD amplification factor (*A*_STD_) vs. irradiation time plot. The plot reports the curve for four representative signals with different magnitude. **d** hsEH CTD—15d-PGJ_2_ interaction epitope mapping. The colour-map reports the percentage *A*_STD_ values as follows: yellow ≤ 33.3%; 33.4% < blue ≤ 66.6%; red > 66.7%

The finding that 15d-PGJ_2_ was able to bind reversibly in the enzyme catalytic pocket was not anticipated, thus saturation-transfer difference NMR (STD-NMR) experiments were performed to support the crystallographic data. The ^1^H-NMR spectrum of the 15d-PGJ_2_ (Fig. 4a) was assigned from deposited chemical shift data^44^ and a ^1^H-NMR spectrum of hsEH CTD was recorded (Supplementary Fig. 6a) as control. The off-resonance (*I*_0_) and on-resonance (*I*_SAT_) spectra (Fig. 4b) were acquired on the hsEH CTD and 15d-PGJ_2_ mixture, in parallel with a control experiment performed by irradiating a solution of prostaglandin alone (Supplementary Fig. 6b). Spectra subtraction of the protein-ligand mixture revealed saturation of several protons of the prostaglandin, and their STD amplification factor (*A*_STD_) values were plotted against the irradiation time, showing the expected hyperbolic profile (Fig. 4c). Epitope mapping indicates a stronger interaction of the di-unsaturated aliphatic chain of 15d-PGJ_2_ with hsEH CTD in solution (Fig. 4d), in agreement with the crystallographic structure. Overall, the combined crystallography and solution approach revealed that 15d-PGJ_2_ is able to bind non-covalently within the catalytic pocket of hsEH CTD.

**Fig. 5:**
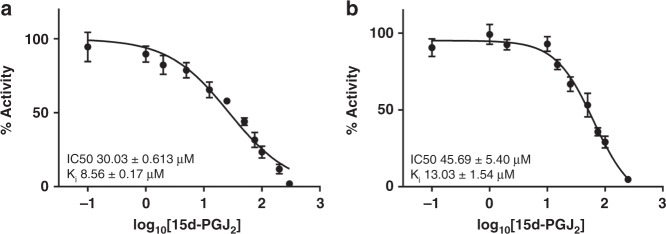
IC50 measurement of 15d-PGJ_2_ for hsEH CTD C423S/C522S (**a**) and WT (**b**). Data presented as average ± SEM of *n* = 6 WT and *n* = 4 C423S/C522S. Supplementary Fig. 7 reports the raw data used to build these IC50 curves as well as the Michaelis–Menten kinetics curves utilised to extrapolate the *K*_M_ value for PHOME. This value was then used in the Cheng–Prusoff equation to obtain *K*_i_ for 15d-PGJ_2_, as described in the methods. *K*_D_ measurements by microscale thermophoresis are reported in Supplementary Fig. 7e and f

Next, we sought to examine the impact of 15d-PGJ_2_ reversible interaction on hsEH enzymatic activity. To assess solely the reversible orthosteric effect of the ligand, the C423S/C522S mutant protein was used in spectrofluorimetric assays, revealing a half maximal inhibitory concentration (IC50) of 30.03 ± 0.61 µM and an inhibitory constant (*K*_i_) of 8.56 ± 0.17 µM (Fig. 5a and Supplementary Fig. 7a). Minimising the incubation time and therefore the formation of covalent adduct(s), the IC50 and *K*_i_ of the 15d-PGJ_2_ for the WT CTD were also measured, as a control (Fig. 5b and Supplementary Fig. 7b). These values (IC50 45.69 ± 5.40 µM; *K*_i_ 13.03 ± 1.54 µM) were largely in agreement with the ones obtained for the C423S/C522S mutant, indicating that both experiments are describing the same molecular process. Based on these data, we concluded that 15d-PGJ_2_ can inhibit hsEH both in the reversible and in the covalent state, in other words both orthosterically and allosterically.

### 15d-PGJ_2_ adduction destabilises the hsEH CTD active site

**Fig. 6:**
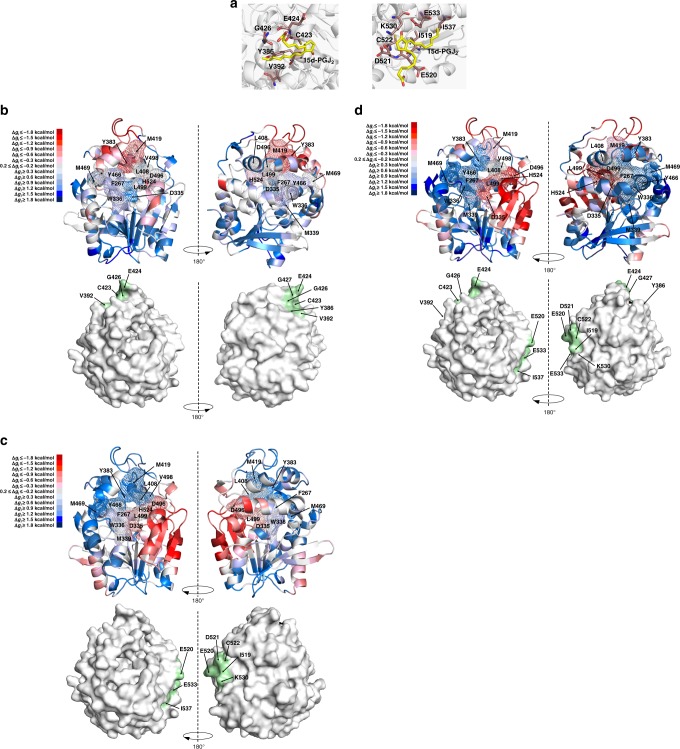
In silico analyisis of the allosteric communication induced by perturbation of the reactive cysteine residues of hsEH CTD. **a** Binding poses of 15d-PGJ_2_ at the predicted C423 (left) and C522 (right) binding sites. The ligand is depicted in yellow, while the interacting residues are depicted in brown. **b**–**d** AlloSigMA analysis of the perturbation of binding sites (C423, C522, C423 and C522 respectively). The top panels show the cartoon model of hsEH CTD, coloured according to the predicted Δ*g*_i_ (blue: destabilization—Δ*g*_i_ ≤ −0.2 kcal/mol; red: stabilisation—Δ*g*_i_ ≥ 0.2 kcal/mol). The bottom panels depict the surface representation, with the perturbed binding sites in green. The full Δ*g*_i_ profiles are reported in Supplementary Fig. 8

Whereas the orthosteric inhibition by 15d-PGJ_2_ was characterised at the atomic level by the crystal structure described above, a molecular description was lacking for the allosteric effect mediated by covalent modifications at C522 and C423. Despite numerous attempts, no satisfactory X-ray crystallography diffraction data for the covalent adducts were obtained. In parallel, we undertook an in silico approach to predict the conformational effects of prostaglandin addition at the two cysteine sites. SwissDock identified several putative binding poses of 15d-PGJ_2_ surrounding C423 and C522. Those with the shortest distance between the ligand electrophilic carbon atoms and the nucleophilic thiols of the reactive cysteine residues were selected (Fig. 6a) and used as input for AlloSigMA, a structure-based statistical mechanical model tool which measures difference in configurational work exerted by binding of an allosteric ligand on a per-residue allosteric free energy (Δ*g*_i_) decomposition, using normal mode analyses^45^. Increased (positive sign) and decrease (negative sign) Δ*g*_i_ correspond respectively to predicted local destabilisation and stabilisation of the configurational energy (Δ*g*_i_) associated with the calculated modes of the ‘rigidified’ (perturbed) binding site. When the C423 site was analysed, a decrease of Δ*g*_i_ was shown for Y383, one of the two epoxide-positioners involved in the hydrolysis (Fig. 6b and Supplementary Fig. 8). Conversely, the catalytic residue D335 exhibited a considerable increase in Δ*g*_i_, suggesting that 15d-PGJ_2_ binding may induce the repositioning of this key residue impairing the catalysis. Moreover, a milder increase in Δ*g*_i_ was observed for the other epoxide positioner Y466, as well as for some of the residues of the W336 niche, suggesting an induced conformational change of this side of the binding pocket upon perturbation of the C423 site. When the C522 site was investigated, the entire catalytic triad (D335, D496, H524) showed a decrease in Δ*g*_i_, whilst the two epoxide positioners (Y383, Y466) and the residues from both W336 niche (W336, M469) and F267 Pocket (F267, L408, M419, V498) exhibited a Δ*g*_i_ increase (Fig. 6c). This would suggest that the ligand binding on the C522 site induces a stabilisation of the catalytic triad, inducing a large conformational change of the binding pocket, and possibly leading to transition towards an inactive state. When both C423 and C522 sites were analysed in conjunction, an enhanced stabilisation of the catalytic triad was associated with a large-amplitude motion of the binding pocket, as showed by the greater destabilisation of the epoxide positioners, as well as the W336 niche and F267 Pocket (Fig. 6d). Altogether, our in silico analysis supports an allosteric regulation of 15d-PGJ_2_ mediated by its covalent binding to the two reactive cysteine residues C423 and C522.

## Discussion

**Fig. 7:**
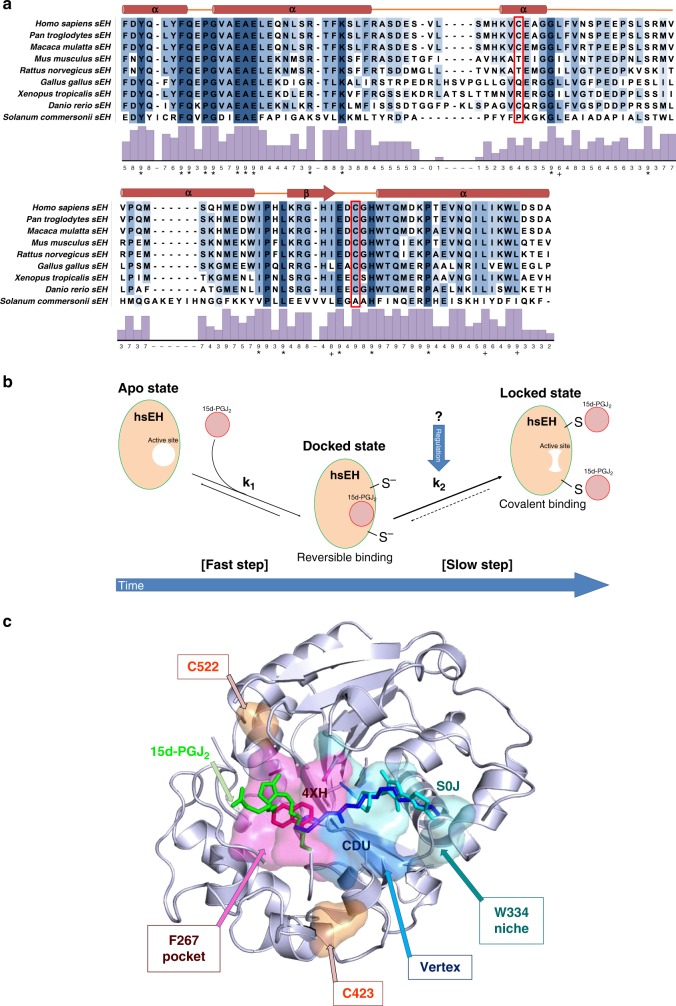
Analysis of sEH binding modes for 15d-PGJ_2_. **a** Protein sequence alignment. Alignment of sEH amino acid sequences of different species, highlighting C423 and C522 in the top and bottom panel respectively. The red boxes highlight the reactive cysteine residues. The secondary structure reported above the alignments refers to the human apo sEH structure. The histograms represent the conservation score calculated by Jalview^71^ (*full conservation, +: full properties conservation). **b** Proposed dock-and-lock binding mechanism. In a first fast step, 15d-PGJ_2_ docks non-covalently in the hsEH CTD binding site (docked state). This is followed by the formation of a covalent complex (locked state), and this step is characterised by a slower kinetics. **c** 15d-PGJ_2_ binding position compared to previously characterised orthosteric inhibitors 4XH, CDU, S0J. The surfaces represent the Phe267 Pocket (magenta), the Vertex (blue) and the Trp334 Niche (cyan), while residues C423 and C522 are reported in orange. The superposition herein reported was performed using PDBs 1EK2^47^, 3WKC^72^ and 5AI6^49^

Previous studies proposed that 15d-PGJ_2_ inhibits murine sEH by covalent modification of C521^30^. In this study, we revealed that 15d-PGJ_2_ exerts inhibition of the human sEH ortholog by binding both non-covalently in the catalytic pocket and covalently to reactive cysteine residues, one of which, namely C423, was not characterised in earlier work. Our X-ray crystallographic structure places 15d-PGJ_2_ in the active site, resulting in reversible enzyme inhibition with a *K*_i_ in the low micromolar range. The position of 15d-PGJ_2_ in the outer region of the F267 Pocket suggests that inhibition is achieved largely by impairment of substrate binding to the active site, although contacts between the ligand and the catalytic residue H524, as well as the epoxy-positioner Y383, do not exclude the possibility of a reduced availability of crucial amino acids involved in the hydrolytic reaction. In addition to this reversible orthosteric inhibition, our studies corroborated that hsEH activity is hindered by covalent modification of reactive cysteine residues, although contrary to the murine enzyme^30^, two residues were unveiled in the human ortholog by our investigations, namely C423 and C522. We demonstrated that, although the covalent modification of a single cysteine site results in inhibition, the adduction to both residues leads to a greater decrease in the enzymatic activity of hsEH CTD. A major outcome of this work is that C423 was discovered as a modification site in human. Interestingly, this residue is conserved only in primates, suggesting that the modulation of soluble epoxide hydrolase differs within the tetrapod lineage (Fig. 7a). Intriguingly, our data appear to indicate that C423 is modified by 15d-PGJ_2_ at faster rate of adduction than C522 and that this leads to a greater level of enzyme inhibition, although the relative importance of these two residues in an in vivo setting remains to be established.

Taken together, our data suggest a dual mode of inhibition of hsEH CTD by 15d-PGJ_2_ (Fig. 7b), that could be modelled as an extension of the ‘dock-and-lock’ mechanism previously described for another 15d-PGJ_2_ target, namely PPARγ^46^. This model envisages that the reversible ‘docking’ of 15d-PGJ_2_ in the catalytic pocket would be followed by the covalent ‘locking’ on the two reactive cysteines C423 and C522. Both states lead to reduction of enzymatic activity, though the fast reversible interaction may facilitate and drive the slower covalent addition (reversible-driven covalent addition), which by its nature would have a prolonged and more potent effect overtime. The position of C423 and especially C522 around the catalytic pocket may support this hypothesis. In this context it may be plausible that the docking-to-locking (or reversible-to-covalent) switch is regulated by the oxidation redox status of the reactive cysteine residues and other environmental clues that may change the reactivity and/or availability of their thiol groups. Nonetheless, a direct and consequential link between the reversible and irreversible mode of inhibition of 15d-PGJ_2_ may not necessarily exist, providing instead alternative ways to inhibit the enzyme.

In our X-ray crystallographic structure, the prostaglandin was found inside the active site, specifically occupying the F267 Pocket of the L-shaped catalytic tunnel. The majority of sEH orthosteric inhibitors are 1,3-disubstituted urea antagonists, and variations thereof, able to position themselves in and around the vertex region, impeding substrate access to the catalytic triad^20,47^ (Fig. 7c). Although numerous sEH antagonist were found to target the F267 region^20,48,49^, we identified a continuous binding surface for 15d-PGJ_2_, involving residues S412, S415, L417 and M419. These unique binding features allow the delineation of a novel interacting surface, at the very entrance of the catalytic site (Fig. 7c), which could potentially be exploited as an anchoring region in the design of new orthosteric hsEH inhibitors with different chemical scaffolds. More importantly though, because of its ability to undergo Michael addition with allosteric residues C522 and C423, 15d-PGJ_2_ could represent a putative lead molecule for the development of new covalent allosteric drugs for hsEH, whose advantages include higher specificity, increased biochemical efficacy, a preference for shallow binding pockets, lower toxicity and lower administration doses, high enough for the drug molecule to reach each target protein and persist until the body has produced more protein^50,51^. Furthermore, the prospect of allosteric inhibition would potentially circumvent the historical drawbacks associated with orthosteric inhibitors, none of which has succeeded in clinical trials, despite intense and sustained efforts^20,21,26,28,29^.

In this study we provide new critical evidence on the mechanism by which the covalent modification of 15d-PGJ_2_ on C522 and C423 inhibits hsEH CTD. Notably, these cysteine residues are not located within the active site, nor are in any way involved in the epoxide hydrolysis, thereby indicating an allosteric modulation of the enzyme via post-translation modification. Our CD studies show that 15d-PGJ_2_ adduction on both C423 and C522 is accompanied by conformational changes in hsEH CTD, possibly affecting the active site. This would in turn disrupt substrate accessibility and/or render substrate binding less favourable thermodynamically. Consistent with this, upon 15d-PGJ_2_ covalent modification the enzyme was no longer capable of binding the high affinity benzylmercaptan compound immobilised onto the BTS resin. The biophysical characterisation was backed up by in silico studies that were able to predict allosteric communication between the C423 and C522 binding surfaces and the catalytic site, particularly affecting the conformation of the two epoxide positioners and various other residues in both W336 niche and F267 Pocket. A picture is emerging in which the covalent modification of these cysteines remote from the catalytic site elicits conformational and dynamics changes of the hsEH CTD towards an inactive state, although the detailed mode of regulation awaits structure determination of C522/C423-adducted covalent complex(es). Notably, in the case of C522, its location in the vicinity of the entrance of the catalytic tunnel does not rule out the possibility that the allosteric regulation may be coupled with a steric hindrance effect also contributing to inhibition. Given its location remote from the catalytic pocket, the same cannot be argued for C423 (Fig. 7c).

From a physiological perspective, it could be envisaged that 15d-PGJ_2_ may regulate hsEH enzyme activity in various tissues where the prostaglandin and the hydrolase co-localise, such as astrocytes, macrophages, and small size arteries^14,17,34^. Interestingly, 15d-PGJ_2_ possesses anti-inflammatory activities^31,34,52^ and its production increases in response to inflammatory stimuli^33^. Although further studies are needed to correlate the levels of 15d-PGJ_2_ production and hsEH activity, one may speculate that enhanced prostaglandin levels may imply a greater inhibition of sEH, which in turn would augment the bioavailability of EETs and other epoxy-fatty acids with anti-inflammatory properties, possibly contributing synergically to the resolution of the inflammation and inducing cardio- and neuroprotection^16,53,54^.

In conclusion, our research unveiled that hsEH CTD undergoes a dual mechanism of inhibition with the endogenous 15d-PGJ_2_, mediated by covalent and non-covalent interactions. The latter reversible interaction uncovered a putative new orthosteric binding surface to be exploited for the design of new drugs. Although the molecular details of hsEH structural perturbation upon covalent adduction are yet to be fully understood, our data taken together strongly support a hitherto undiscovered allosteric mechanism of hsEH regulation. Altogether, this study provides new insight in the regulatory mechanism of the epoxy-hydrolytic activity of hsEH and paves the way for new modes of sEH inhibition for improving cardiovascular health, inflammation and diabetes.

## Methods

### Plasmid construction and site directed mutagenesis

The EPHX2 (T230-M555—hsEH CTD) cDNA was cloned in the bacterial expression vector pET3a, as described previously^41^. Single mutants C423S and C522S were generated with the Q5^®^ Site-Directed Mutagenesis Kit (NEB). The double mutant C423S/C522S (C423S/C522S) was produced through two subsequent cycles of mutagenesis using the same kit. The mutagenic primers (Sigma), were designed with the NEB changer webtool according to the kit specifications (C423S_R: 5′GCATAAAGTCAGTGAAGCGGG3′; C423S_F: 5′ATGGATAAAACACTCTCATCG3′; C522S_R: 5′CATTGAGGACAGTGGGCACTG3′; C522S_F: 5′TGTCCCCTTTTCAGGTGG3′). Successful mutagenesis was confirmed by sequencing (Eurofins MWG).

### Protein expression and purification

The hsEH CTD and its mutants were expressed in Ros2^TM^(DE3) (EMD Millipore) and purified as described previously for WT^41^.

### Electrospray ionisation mass-spectrometry (ESI-MS)

hsEH CTD protein sample was dialysed overnight at 4 °C in 25 mM HEPES, 300 mM NaCl, 10% glycerol, pH 7.4, 10 μM tris(2-carboxyethyl)phosphine (TCEP) (reaction buffer). The protein was incubated overnight at 4 °C 1:8 v/v (molar ratio) with 15d-PGJ_2_ (BertinPharma—dissolved in PBS), AUDA (Sigma—dissolved in PBS), and PBS as control. At the end of the incubation period, the reaction was quenched by adding 5 mM DTT, and excess ligand was removed by buffer exchange on an Amicon Ultra-0.5 mL centrifugal Filter (*Merck*) at 10,000×*g* and 4 °C. The samples were diluted in 0.1% formic acid at a final concentration of 60 μg mL^−1^, and injected directly into the maXis instrument (Bruker) via a motorised pump with at a flow rate of 10–50 μL min^−1^. The spectra were recorded for 3–30 min at a spectral rate of 1 Hz, with an end plate offset of −500 V, a capillary voltage of −4500 V, a nebuliser pressure of 3.0 bar, and a tuned ISCID energy between 80 and 110 eV. The spectra obtained were deconvoluted using microTOF software (Bruker), performing a maximum entropy analysis.

### Liquid chromatography-tandem mass spectrometry (LC-MS/MS)

Fifty microgram of hsEH CTD, incubated overnight at 4 °C with 15d-PGJ_2_ (1:8 v/v protein-to-ligand molar ratio), was separated by SDS-PAGE on a 20% bis-acrylamide gel, for 30 min at 100 V (according to Laemmli protocol^55^). The protein bands were cut in discrete pieces and digested overnight with 5 μg of trypsin (Sigma-Aldrich) at room temperature, after an initial incubation at 37 °C for 2 h. Peptides were extracted from the gel by a series of acetonitrile and aqueous washes, and the extract was lyophilised and resuspended in 10 μL of 50 mM ammonium bicarbonate. Chromatographic separation of the peptides was performed using an EASY NanoLC system (ThermoFisher Scientific), by reversed phase chromatography on a 75 μm C18 column, using a three-step linear gradient of acetonitrile in 0.1% formic acid. The gradient was delivered to elute the peptides at a flow rate of 300 nL min^−1^ over 60 min. The eluate was ionised by electrospray ionisation using an Orbitrap Velos Pro (ThermoFisher Scientific), operating under Xcalibur v2.2. The instrument was programmed to acquire in automated data-dependent switching mode, selecting precursor ions based on their intensity for sequencing by collision-induced fragmentation using a Top20 CID method. MS/MS analyses were conducted using collision energy profiles that were chosen based on the mass-to-charge ratio (*m/z*) and the charge state of the peptide. Raw mass spectrometry data were processed into peak list files using Proteome Discoverer (ThermoScientific; v1.4). Processed raw data were analysed using the Mascot 2.2.06 (Matrix Science) search algorithm against ‘All Taxonomy' in the Uniprot database and an in-house database containing the protein sequence of interest, assuming the digestion enzyme trypsin. Mascot was searched with a fragment ion mass tolerance of 0.80 Da and a parent ion tolerance of 20 ppm. 15d-PGJ_2_ modification of cysteine residues was specified in Mascot as variable modifications. Scaffold 4.7.5 (Proteome Software Inc) was used to validate MS/MS based peptide and protein identifications. High stringency filters of 95% confidence interval (CI) for minimum protein and 0% CI for peptide values was applied. A 95% probability CI in the MOWSE scoring algorithm in Mascot was applied. Protein probabilities were assigned by the Protein Prophet algorithm^56^. The analysis yielded 42 unique peptide fragments (88% sequence coverage), of which two were unambiguously modified by 15d-PGJ_2_.

### High-pressure liquid chromatography (HPLC) analyses

hsEH CTD WT, C423S, C522S and C423S/C522S proteins were dialysed overnight at 4 °C in reaction buffer (see ESI-MS section), and then incubated at 4 °C 1:8 v/v (molar ratio) with 15d-PGJ_2_ (dissolved in PBS). After 30 min, 2.5 h or 20 h, the reaction was quenched by adding 5 mM DTT and immediately flash-frozen in liquid nitrogen. 2.5 μg of the protein samples were mixed with 10 mM TRIS-HCl pH 7.5, to be loaded, using an Agilent 1200 HPLC system, on a reversed phase column (ACE 5 C18–300, 250 × 4.6 mm). The species were eluted with a 48–60% gradient of acetonitrile and 0.1% TFA, over 12 min at 1 mL min^−1^. The traces obtained were deconvoluted with Fityk software^57^ and the peaks integrated with a Pearson VII function. The areas under the peaks were used to back calculate the amount of adduct observed at each time point of the reaction and extrapolate the rate of adduction.

### Purification of covalent adducts

To purify the covalent adducts, we designed a tailored Benzylthio-Sepharose (BTS) affinity chromatography protocol. Apo-hsEH CTD binds BTS via interactions between the enzyme’s hydrophobic binding pocket and benzylmercaptan moieties immobilised onto Sepharose beads^40,41^, whilst covalently adducted counterparts do not interact with the resin and were collected in the column flow-through fractions. 15d-PGJ_2_-hsEH CTD covalent adducts were prepared as follows. 0.5 mg of WT, C423S, C522S, and C423S/C522S proteins were dialysed overnight at 4 °C in 25 mM 3-(N-morpholino)propanesulfonic acid (MOPS) pH 7.4, 75 mM NaCl, 5% glycerol, 10 μM TCEP (binding buffer), and then incubated at 4 °C 1:8 v/v (molar ratio) with 15d-PGJ_2_ (dissolved in PBS) or binding buffer (as control). After overnight incubation, the mixtures were loaded onto 500 μL of BTS resin in gravity columns (prepared in-house^41^), previously equilibrated in binding buffer. After 15 min incubation at room temperature, the flow-through was collected and the resin washed five times with 500 μL of binding buffer. Five hundred microliter of elution buffer (binding buffer containing 1 mM 2-benzoyl-3-phenyloxirane) were added onto the resin to incubate for 15 min before elution. The protein samples were eluted with 5 × 500 μL elution buffer and collected. SDS-PAGE analyses were carried out to identify the proteins in the fractions. The flow-through and elution fractions containing protein were pooled together and dialysed overnight at 4 °C in 20 mM TRIS-HCl pH 7.4, 100 mM NaCl, 2% glycerol, 10 μM TCEP, concentrated to 0.5 mg mL^−1^ and flash frozen in liquid nitrogen.

### hsEH CTD enzymatic activity measurements

The activity of the isolated proteins was measured using a spectrofluorometric method which monitors hsEH-mediated hydrolysis of the synthetic substrate 3-phenyl-cyano(6-methoxy-2-naphthalenyl)methyl ester-2-oxiraneacetic acid (PHOME), through the detection of the final fluorescent product 6-methoxy-2-naphthaldehyde (6M2N)^58^. Protein samples were diluted to a final concentration of 50 nM in 25 mM TRIS-HCl pH 7.4 in a black 96-well polystyrene microtiter plate (Thermo Scientific). A fresh 0.4 mM solution of the synthetic substrate PHOME was prepared in DMSO and diluted 1:40 v/v in the 96-well plate. The measurements of fluorescence units (RFUs) generated by the hsEH CTD-mediated hydrolysis of PHOME were carried out in quadruplicate for 20 min using a POLARstar Omega (BMG Labtech), with the following setup: excitation/emission wavelengths 330/460 nm, gain 750, detection every 45 s, and temperature at 30 °C. Each sample of three different protein preparations was tested in triplicate. Quantification of the epoxy-hydrolytic rate was carried out using a conversion curve reporting the fluorescence of 6M2N, as a function of the fluorophore concentration, as described previously^41^.

### Ultraviolet–visible (UV) and circular dichroism (CD) spectroscopy

UV and CD spectra were acquired at 25 °C on an Applied Photophysics Chirascan Plus spectrometer (Leatherhead, UK). 10 mm (500–220 nm) and 0.5 mm (260–190 nm) Quartz Suprasil rectangular cells (Starna Scientific Ltd) were employed. The instrument was flushed continuously with pure evaporated nitrogen throughout the experiment. The following parameters were set: 2 nm spectral bandwidth, 1 nm step size, and 1 s instrument time per point. Light-scattering adjustment and buffer subtraction were applied to the UV spectra of each sample, using the Chirascan Pro-Data Software (APL). UV and CD spectra of the covalent adducts were recorded at a final concentration of 0.15 mg mL^−1^ (calculated using the *A*_280nm_ value obtained upon light-scattering correction and the extinction coefficient computed using the ProtParam tool within ExPASy Portal^59^). The far-UV CD spectra were smoothed with a window factor of 4 using the Savitzky-Golay method for better presentation, then corrected for concentration and pathlength, and expressed in terms of mean residue ellipticity (MRE). Protein secondary structure content was assessed using the SELCON 3 algorithm (data set 4), embedded in the Dichroweb webtool^60^.

### X-ray crystallography

Recombinant hsEH CTD was crystallised by vapour diffusion, in sitting drops containing 100 nL of protein solution (hsEH CTD concentration 10 mg mL^−1^, in 50 mM HEPES, 300 mM NaCl, 10% glycerol, 3 mM DTT, pH 7.4), and 200 nL of precipitation buffer (22.5% PEG 3350, 0.2 M malic acid), equilibrated against a 20 μL reservoir of precipitant buffer at 4 °C. After ~5 days, cubic crystals of around 30 × 30 × 30 μm were obtained. Following soaking in precipitant buffer containing 25% ethylene glycol and flash cooling in liquid nitrogen, crystals yielded diffraction data to 2.6 Å at the Diamond Light Source, Didcot, UK. The diffraction data were reduced, scaled, and merged using XIA2 software^61^. The crystals belonged to space group *I*_121_, with unit cell parameters *a* = 88.22, *b* = 80.14, and *c* = 104.70 Å. Crystals were then soaked in precipitation buffer containing 10 mM 15d-PGJ_2_ (Cayman Chemical, USA), incubated for 15 min, cryoprotected with 25% ethylene glycol and flash frozen in liquid nitrogen. Diffraction yielded a resolution of 2 Å. Upon reduction, merging, and scaling with XIA2 software^61^, the structure was solved by molecular replacement in PHENIX^62^, using the deposited PDB file 3ANS^63^. The density map was explored and built in COOT^64^, and the structure was refined iteratively by automatic structure refinement using the software Refmac5 within CCP4 suite^65^. The 15d-PGJ_2_ molecule was added into the final stages of refinement. Data reduction and refinement statistics are reported in Table 2.

### Half maximal inhibitory concentration (IC50) measurements

The inhibitory potency of 15d-PGJ_2_ was measured using an end-point adaptation of the PHOME spectrofluorometric method described above. Recombinant hsEH CTD WT and C423S/C522S were reduced with 10 µM TCEP on ice for 15 min, then diluted in a 96-well polystyrene microtiter plate in freshly prepared 25 mM TRIS-HCl pH 7.4 to a final concentration of 15 nM. The proteins were then incubated at room temperature for 10 min with 15d-PGJ_2_ (diluted 1:40 v/v to obtain increasing concentration between 0.1 and 250 μM). A fresh 0.4 mM solution of PHOME was prepared in DMSO and diluted 1:40 v/v in the 96-well plate. A POLARstar Omega (BMG Labtech) was used to measure the relative fluorescence units (RFUs) for 20 min, using the same setting as above. Measurements were performed in four replicates, for a total of four technical repeats. The readouts at 12 min reaction times were corrected for the background signal generated by the PHOME auto-hydrolysis, converted into nmol of 6M2N (6-methoxy-2-naphthaldeyde) and plotted as percentage of activity against the Log_10_ of 15d-PJG_2_ concentration^58^. The data points were fitted with ‘log(inhibitor) vs. response—variable slope' in GraphPad, and further considered only if *R*^2^ was ≥0.9. Inhibitory constants (*K*_i_) values were calculated using the Cheng-Prusoff equation^66^.

### Saturation-transfer difference NMR (STD-NMR)

STD-NMR is a technique for the detection of reversible binding interactions between small molecule ligands and macromolecular interactors in solution^67^. NMR spectra were recorded on a Bruker AVANCE-500 NMR spectrometer (Bruker). Both hsEH CTD and 15d-PGJ_2_ samples were prepared in 50 mM HEPES, 300 mM NaCl, 10% glycerol, 0.1 mM TCEP, pH 7.4, 10% D_2_O. After recording the ^1^H-NMR-I_0_ spectra of both hsEH CTD (10 μM) and 15d-PGJ_2_ (1000 μM), STD-NMR experiments were performed at 293.15 K, mixing protein and ligand at a 1/100 v/v molar ratio, with the pulse sequence stddiffesgp.3, using a train of Gaussian pulses of 50 ms duration at a power level of 86 Hz, spaced by a delay of 1 ms. The irradiation power of the selective pulses was set to (γ/2π)B1 = 86 Hz. Selective pre-saturation of the protein was achieved by a train of Gauss-shaped pulses of 50 ms length each, separated by a 1 ms delay. Irradiation was performed at −0.55 ppm (and −30 ppm as reference) for a total saturation time spanning between 0.5 and 6 s. The subtraction of the saturated spectra from the reference spectra was performed using TopSpin3.2, after identical processing and phasing. Control experiments were performed by irradiating the ligand in isolation (same experimental setting as for above): no STD signal was detected, confirming that the saturation observed in presence of the protein was due to the interaction between hsEH CTD and 15d-PGJ_2_. The STD effect was quantified in terms of amplification factor (*A*_STD_), calculated with the following equation:

$$A_{{\mathrm{STD}}}{\mathrm{ = }}\frac{{I_{{\mathrm{STD}}}}}{{I_0}} \cdot \frac{{[{\mathrm{15dPGJ}}_2]}}{{{\mathrm{[hsEH}}\;{\mathrm{CTD]}}}}$$

where *I*_STD_/*I*_0_ corresponds to the ratio between STD (*I*_STD_) and off-resonance (*I*_0_) signals,  and [15d-PGJ_2_]/[hsEH CTD] is the ligand-to-protein molar ratio^68^. Percentage *A*_STD_ values were calculated by normalising for the greatest *A*_STD_ value observed (C20) and used to model the hsEH CTD—15d-PGJ_2_ epitope mapping interaction^68^.

### Microscale thermophoresis

hsEH CTD protein was dialysed in 25 mM HEPES, pH 7.4, 300 mM NaCl, 2% glycerol, 10 µM TCEP and labelled in dark with the amino-reactive blue dye, following the Monolith NT Protein Labelling kit protocol (NanoTemper Technologies). Hundred nanomolar of labelled enzyme were incubated for 10 min at room temperature with 15d-PGJ_2_ at concentrations ranging between 0.0038 and 125 µM (1:1 serial dilution). Microscale thermophoresis (MST) experiments were performed at 20% of LED power and 40% of MST power with a Monolith NT.115 (NanoTemper Technologies). Data analysis was performed using the MO.control software (NanoTemper Technologies), fitting the auto-normalised data in manual mode. *K*_D_ values were obtained by fitting with dose-response equations implemented in the MO.control software.

### AlloSigMA analyses

AlloSigMA is a structure-based statistical mechanical model tool which estimates the per-residue allosteric free energies (Δ*g*_i_) associated with an effector binding. Particularly, Δ*g*_i_ > 0 and Δ*g*_i_ < 0 correspond to increased and decreased free energies associated to changes in stability due to allosteric signalling. More precisely, these values quantify variations in configurational work exerted on a specific residue, as a consequence of changes in the measured configurational ensemble upon ligand binding (by normal mode analysis). The ligand binding process is simulated in this framework by rigidifying (‘perturbing’) the residues involved in the binding. The free energy variation associated with such perturbation is approximated by a summation over the normal modes^45^. AlloSigMA analyses were carried out after identifying putative 15d-PGJ_2_ binding sites around the two reactive cysteine residues C423 and C522 using SwissDock^69^. The docking of 15d-PGJ_2_ (ZINC05972962) was performed in accurate mode on the apoprotein crystal structure solved in this study, centring the docking region of interest on the Chain A sulphur atoms of C423 and C522, and allowing 3 Å side chains flexibility. The poses were further considered only when the ΔG obtained was lower than −5 kcal mol^−1^. Once the binding sites were defined, the per-residue allosteric free energy (Δ*g*_i_) calculations were carried out using the AlloSigMA web-server^70^, perturbing the putative C423 and C522 binding sites (in isolation and together). The results were presented in graphics coloured in red and blue scales to indicate, respectively, stabilised and destabilised residues.

### Statistics and reproducibility

The ESI-MS experiments, HPLC purifications, UV and CD analyses, IC50 values measurements, STD-NMR experiments, MST analyses, and enzymatic activity measurements were carried out in at least three biological replicates. The statistical significance for the enzymatic activity experiments was obtained by comparing the enzymatic rates with one-tailed homoscedastic *t*-test and reported in plots as average ± SEM. The statistical significance was reported as follows: *0.05 < *p* ≤ 0.005, **0.005 < *p* ≤ 0.0005, ***p < 0.0005.

### Reporting summary

Further information on research design is available in the Nature Research Reporting Summary linked to this article.

## Supplementary information

Supplementary information

Supplementary data

Reporting Summary

## Data Availability

Atomic coordinates for the X-ray crystallographic structures of hsEH CTD and hsEH CTD-15d-PGJ_2_ complex are deposited in the Protein Data Bank under the accession codes 6I5E (apo) and 6I5G (complex). All relevant data within the manuscript are available for review or discussion by interested parties. Additional data are included as Supplementary Information. If necessary, we can facilitate experiments planned by other parties that are related to the results of our study. There are no large datasets and accession codes that have contributed to the information in this paper. All relevant data supporting the findings of this study are available from the authors upon reasonable request.
