# Supplementary material for: 15-deoxy-Δ12,14-Prostaglandin J2 inhibits human soluble epoxide hydrolase by a dual orthosteric and allosteric mechanism
Source: Commun Biol. 2019 May 17;2:188. doi: 10.1038/s42003-019-0426-2 (PMC6525171; doi:10.1038/s42003-019-0426-2)
Supplement: Supplementary file 1 — Supplementary information [file 42003_2019_426_MOESM1_ESM.pdf]

Supplementary Figures

Supplementary Figure 1

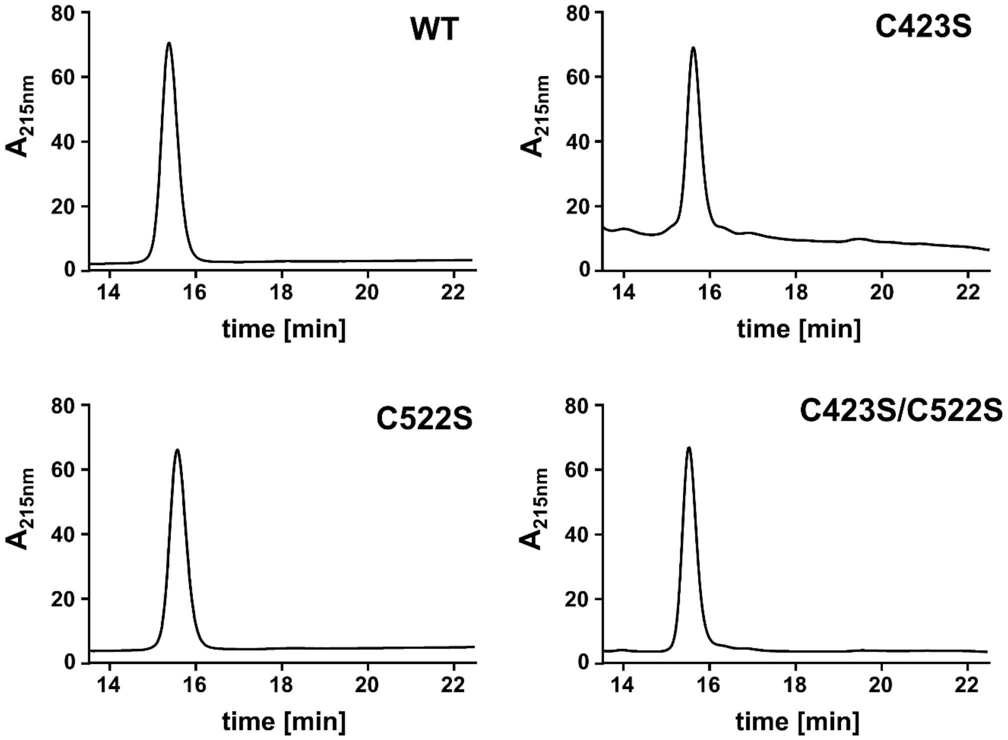

Supplementary Fig. 1 hsEH CTD WT, C423S, C522S, and C423S/C522S proteins were dialysed overnight at 4ºC in 25 mM HEPES pH 7.4, 300 mM NaCl, 10% glycerol, 10 µM TCEP, and incubated at 4ºC with PBS. After overnight incubation, 7.5 µg of the protein samples were mixed in 500 µL of 10 mM TRIS-HCl pH 7.5 and loaded onto a reversed phase column (ACE 5 C18-300, 250 × 4.6 mm), using an Agilent 1200 HPLC system (see Methods section for further details).

Supplementary figure 2

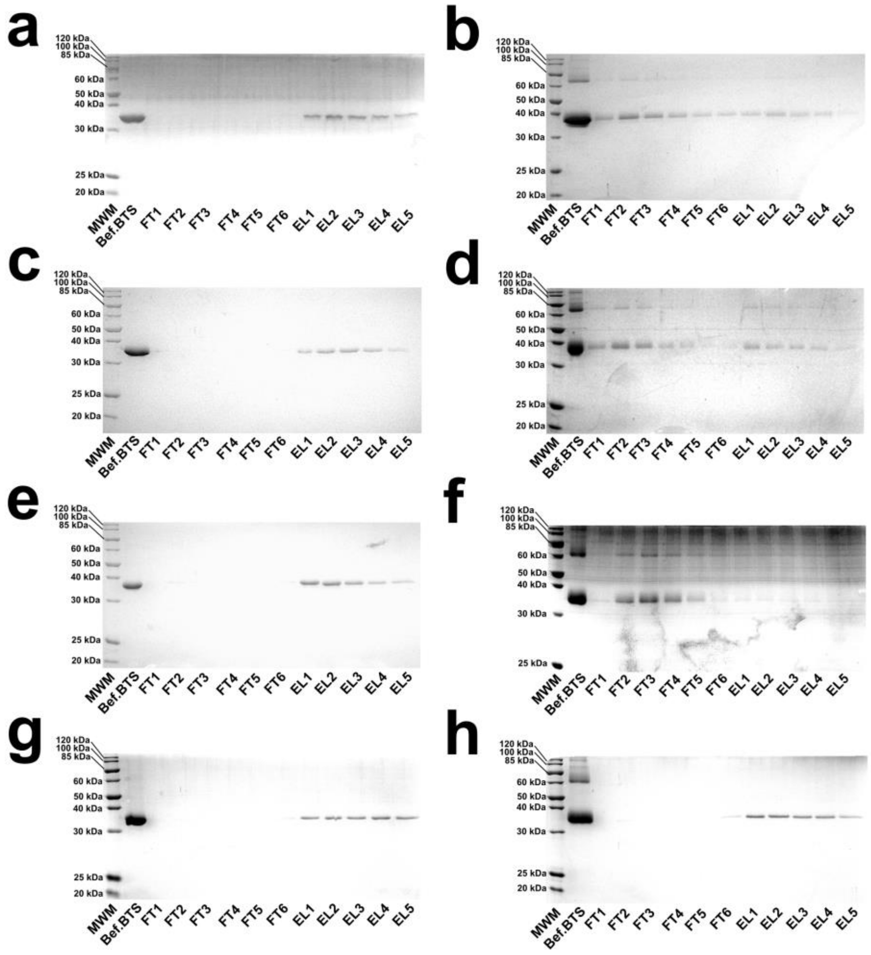

Supplementary Fig. 2 Full uncropped SDS-PAGE of purifications of 15d-PGJ_2-hsEH CTD covalent adducts. **a** hsEH CTD WT buffer treated; **b** hsEH CTD WT 15d-PGJ_2 treated; **c** hsEH CTD C423S buffer treated; **d** hsEH CTD C423S 15d-PGJ_2 treated; **e** hsEH CTD C522S buffer treated; **f** hsEH CTD C522S 15d-PGJ_2 treated; **g** hsEH CTD C423S/C522S buffer treated; **h** hsEH CTD C423S/C522S 15d-PGJ_2 treated. (MWM: molecular weight marker, Bef. BTS: before BTS purification; FT: flow-through fraction; EL: elution fraction).

Supplementary figure 3

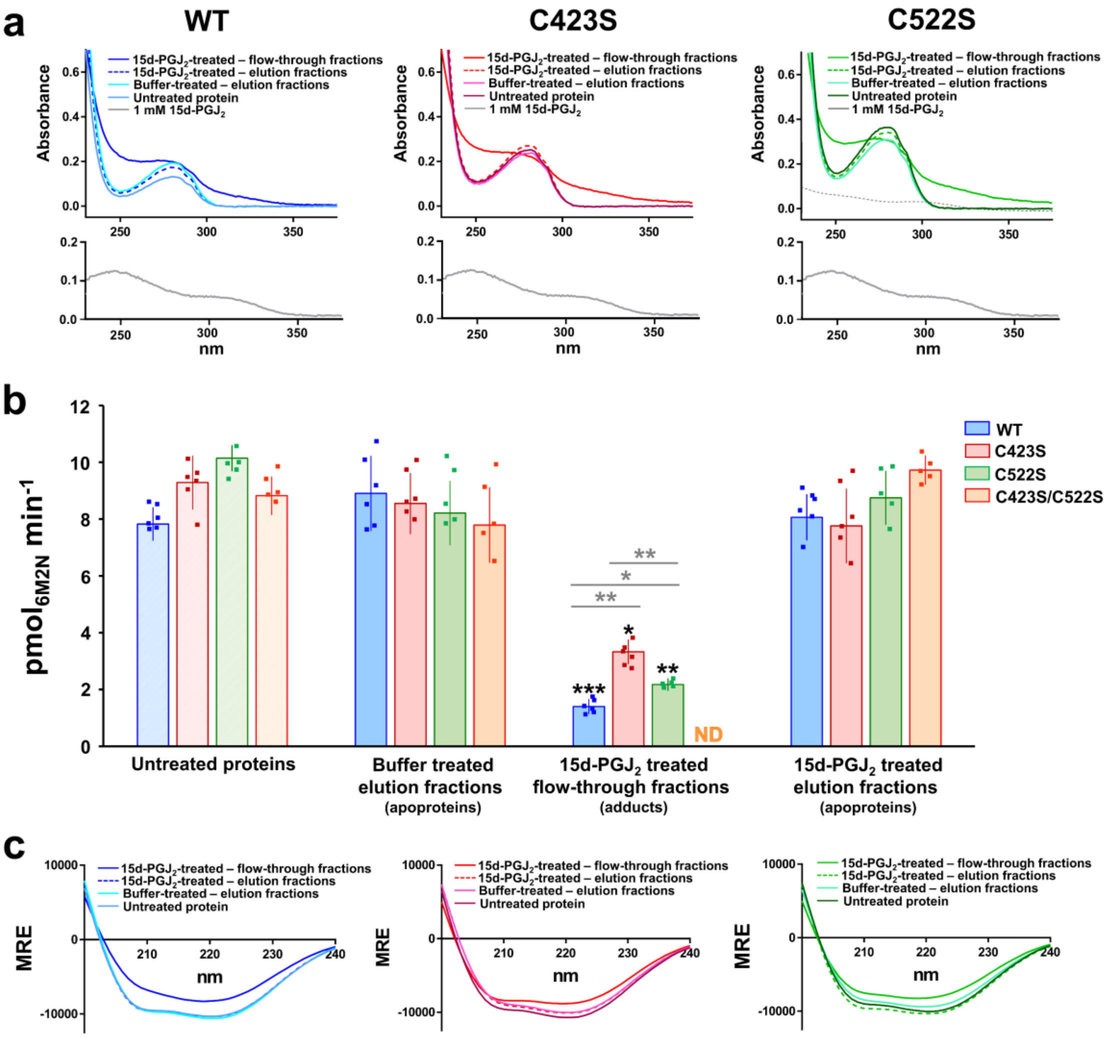

Supplementary Fig. 3 Analysis of hsEH WT, C522S, C423S and C522SC423S proteins and their covalent modification by 15d-PGJ2. **a** UV analyses of all the fractions separated in the BTS purifications compared with controls (protein and 15d-PGJ2 alone). The profile of the flow-through fractions is consistent with 15d-PGJ2-modified species, showing UV absorbance peaks at 280, 250 and 330 nm. None of the other protein fractions gave a UV signal at 250 and 330 nm, indicating that they were not covalently adducted by the prostaglandin. **b** Enzymatic activity of all of the fractions separated in the BTS purifications. The covalently modified WT, C423S and C522S proteins (collected in the BTS flow-through fractions) exhibited reduced enzymatic activity compared to the respective apoproteins (eluted from the BTS resin) and controls (untreated proteins and proteins eluted from the BTS resin upon treatment with buffer alone). This demonstrated that reduction of the enzymatic activity of the covalent adducts was linked to the covalent modification of C522 and C423 by 15d-PGJ2. Data presented as average ± SEM of *n* = 6 WT, *n* = 6 C522, *n* = 5 C423S, and *n* = 5 C423S/C522S. (Source data available in Supplementary Data 2). **c** CD analyses of all the fractions separated in the BTS purifications. The comparison of the far-UV CD spectra of all of the fractions separated revealed a conformational change upon 15d-PGJ2-mediated adduction.

Supplementary figure 4

The details of the unit cell dimerisation were analysed using the protein interface surfaces and assemblies (PISA)[1] tool in CCP4[2]. The model revealed that two hsEH CTD units interacted forming a homodimer in the crystallographic symmetry. The analysis computed a monomer interface formed by 26 amino acids (7.9%), and an overall area of 725.6 Å^2. PISA detected ten hydrogen bonds and six salt bridges, for an overall binding energy of -8 kcal/mol. The PISA analysis calculated a probability under 30% for the hsEH CTD dimer formation, suggesting that the interactions predicted do not result in the formation of the quaternary structure in solution. The prediction was in agreement with previously reported SEC results[3], where hsEH CTD was isolated monodispersed and monomeric in solution.

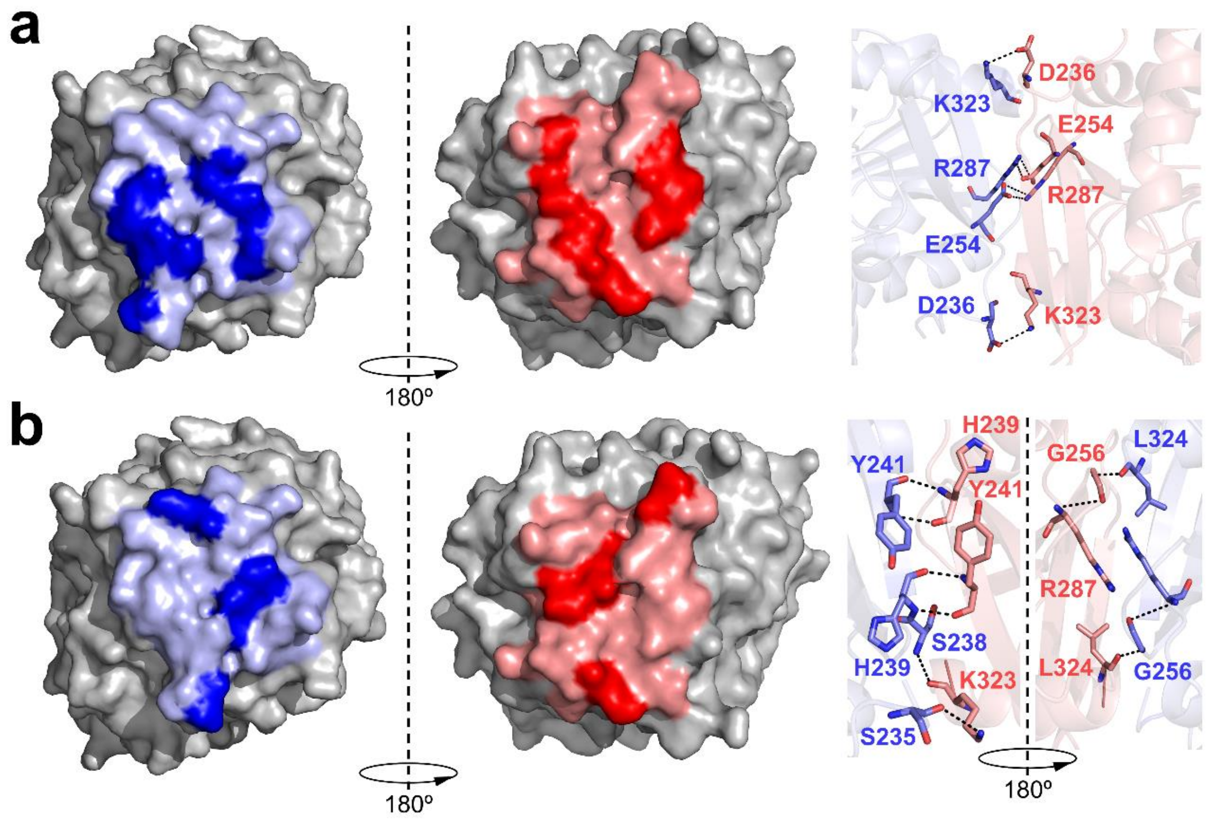

Supplementary Fig. 4 Crystallographic dimer interactions. The light-colored surface indicates the whole dimerisation interface, while the dark surface designates the amino acids implicated in the bonds formation. **a** Hydrogen bonds between chains A and B. **b** Salt bridges between chains A and B.

Supplementary figure 5

BLAST-like dynamic amino acids sequence alignments were performed using the software PyMOL[4]. BLAST-like BLOSUM62-weighted dynamic amino acids alignment analyses[5] were carried out with 25 refinement cycles to improve the fit.

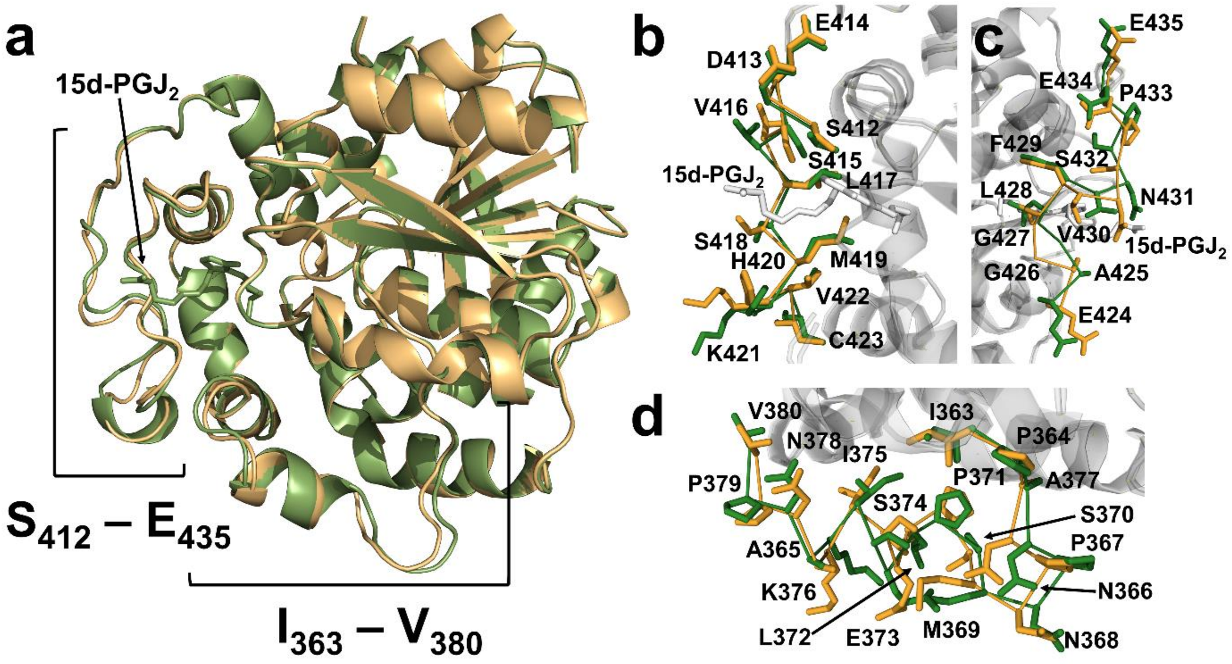

Supplementary Fig. 5 hsEH CTD conformational changes upon 15d-PGJ$_2$ binding. **a** Superposition of the structures of apo vs. complex (shown in orange and green respectively). The alignment revealed a conformational change in the S412-E435 loop and in the I363-V380 helix. **b** Zoom-in of the S412-C423 region. The side chains of amino acids such as V416, S415, H420, and K421 were reoriented upon binding. **c** Zoom-in of the E424-E435 region. The ligand interaction induced a shift and side chain repositioning of most of the amino acids of this region. **d** Zoom-in of the I363-V380 region. The short loop-α-helix underwent conformational change upon 15d-PGJ$_2$ binding. The amino acids side chains of the short α-helix P367-P379 were reoriented.

Supplementary figure 6

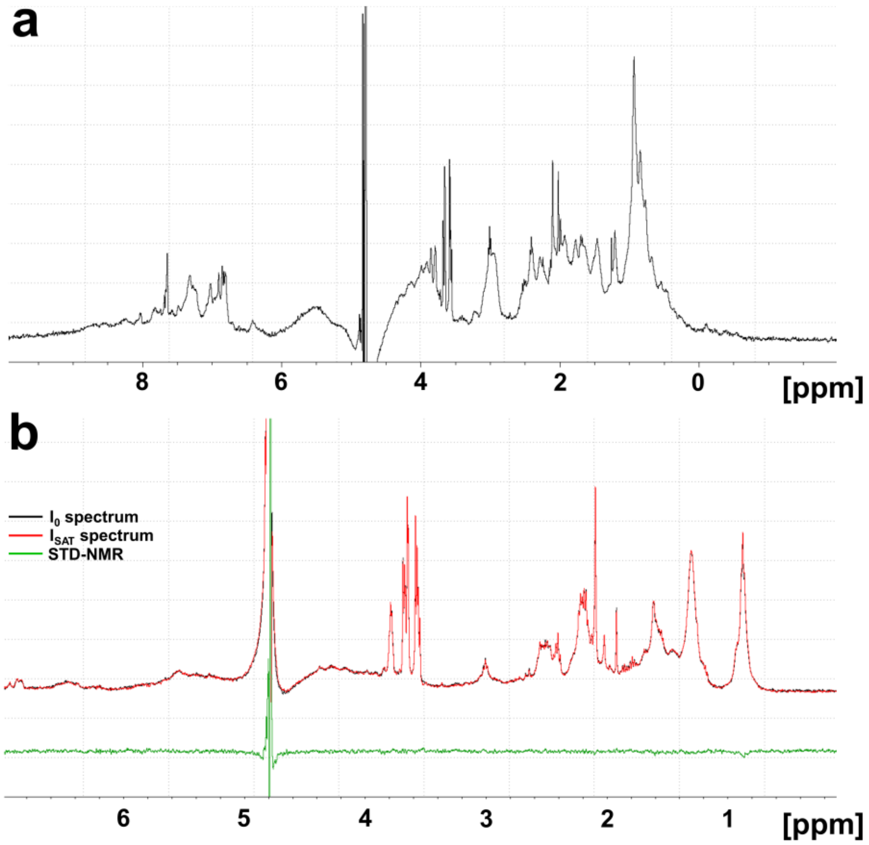

Supplementary Fig. 6 Saturation-transfer difference nuclear magnetic resonance (STD-NMR) control experiments. **a** ^{1}H-NMR spectrum of 10 μM hsEH CTD. **b** Comparison between I_0 (black) and I_{SAT} (red) spectra of 15d-PGJ$_2$. The spectrum reports the effects of the irradiation at -0.55 ppm for 6 seconds on the signal of the prostaglandin in isolation. No STD signal was observed (green), confirming that the chosen frequency was suitable to perform STD-NMR experiments in the presence of hsEH CTD.

Supplementary figure 7

Analysis of Michaelis–Menten kinetics was performed evaluating the reaction rate as a function of increasing substrate concentrations. Recombinant hsEH CTD was reduced with 10 µM TCEP for 15 minutes on ice and diluted in a 96-well polystyrene microtiter plate in freshly prepared 25 mM TRIS-HCl pH 7.4 to a final concentration of 5 nM. DMSO stock solutions of PHOME were prepared in a range of concentrations between 0.25 and 25 µM and diluted 1:40 v/v in the plate. A POLARstar Omega (BMG Labtech) spectrophotometer was used to measure the relative fluorescence units (RFUs) for 20 min, using the same settings as in the Methods. RFU values were corrected for the background signal generated by the PHOME auto-hydrolysis and converted into $nmol_{6M2N}$/min using a conversion curve as in [3]. The initial velocities were then obtained by regression of the linear portion of the curves (first 12 minutes) and plotted against the PHOME concentration. With GraphPad, the data points were fitted with a Michaelis–Menten equation, yielding the K_M value. The fitting accepted when R^2 was > 0.9. The K_M value of PHOME was used to calculate the K_i for 15d-PGJ_2, as described in the Methods. We also measured a dissociation constant (K_D) using microscale thermophoresis, as described in the Methods.

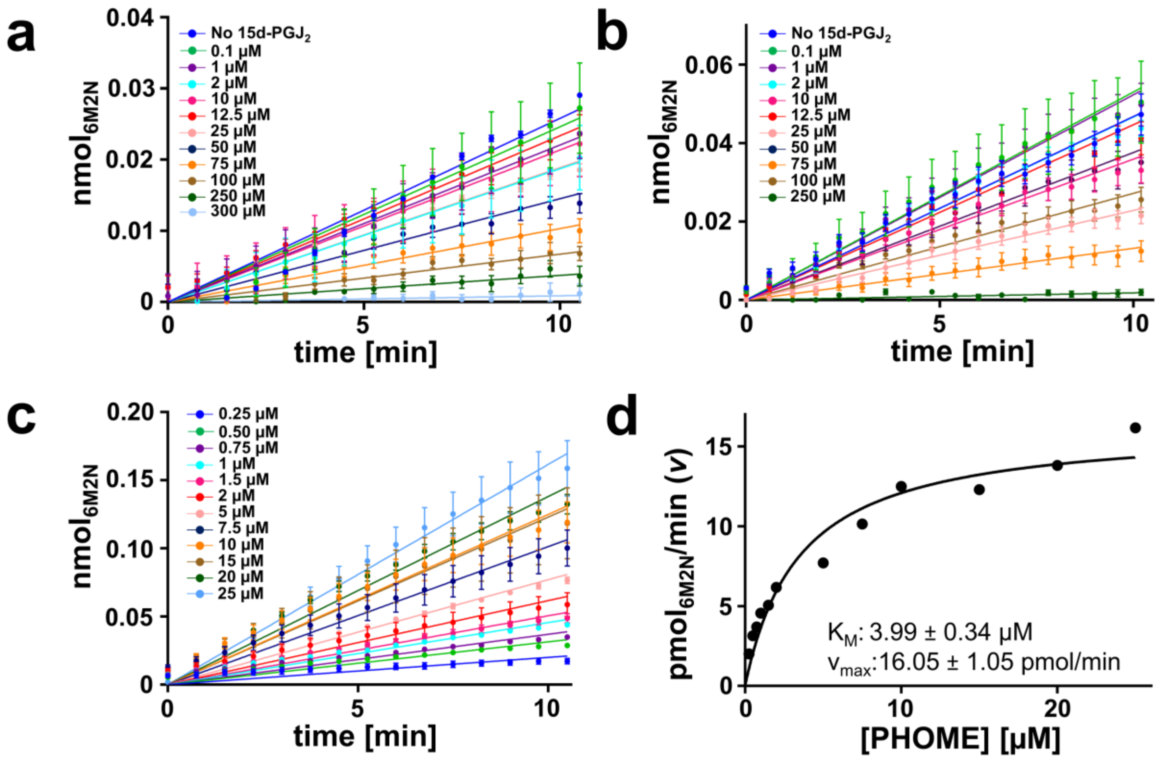

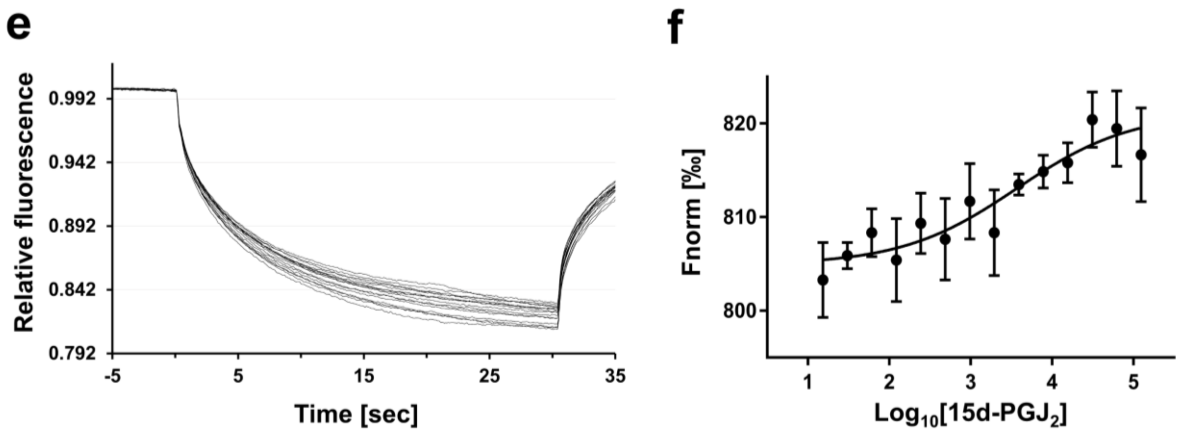

Supplementary Fig. 7 Determination of kinetic and inhibition of hsEH CTD. **a-b** Representative IC50 raw data plots for 15d-PGJ_2 measured with hsEH CTD C423S/C522S (a) and hsEH CTD WT (b). The legends report the final 15d-PGJ_2 concentrations used in the assay. **c** Representative Michaelis–Menten kinetics raw data of hsEH CTD WT. The legend reports the final PHOME concentrations used in the assay. **d** Representative Michaelis–Menten kinetics plot of the catalysis of PHOME substrate performed by hsEH CTD WT. The K_M and v_{max} values are reported as average ± SEM of n = 4. **e** Representative Microscale thermophoresis (MST) trace of 15d-PGJ_2 binding reversibly to hsEH CTD WT. **f.** T-jump signal plot. Data points were fitted with dose-response curves, obtaining the affinity constant of binding of 15d-PGJ_2 to hsEH CTD WT of 3.683 ± 1.081 µM. Data reported as average ± SEM of n = 3.

Supplementary figure 8

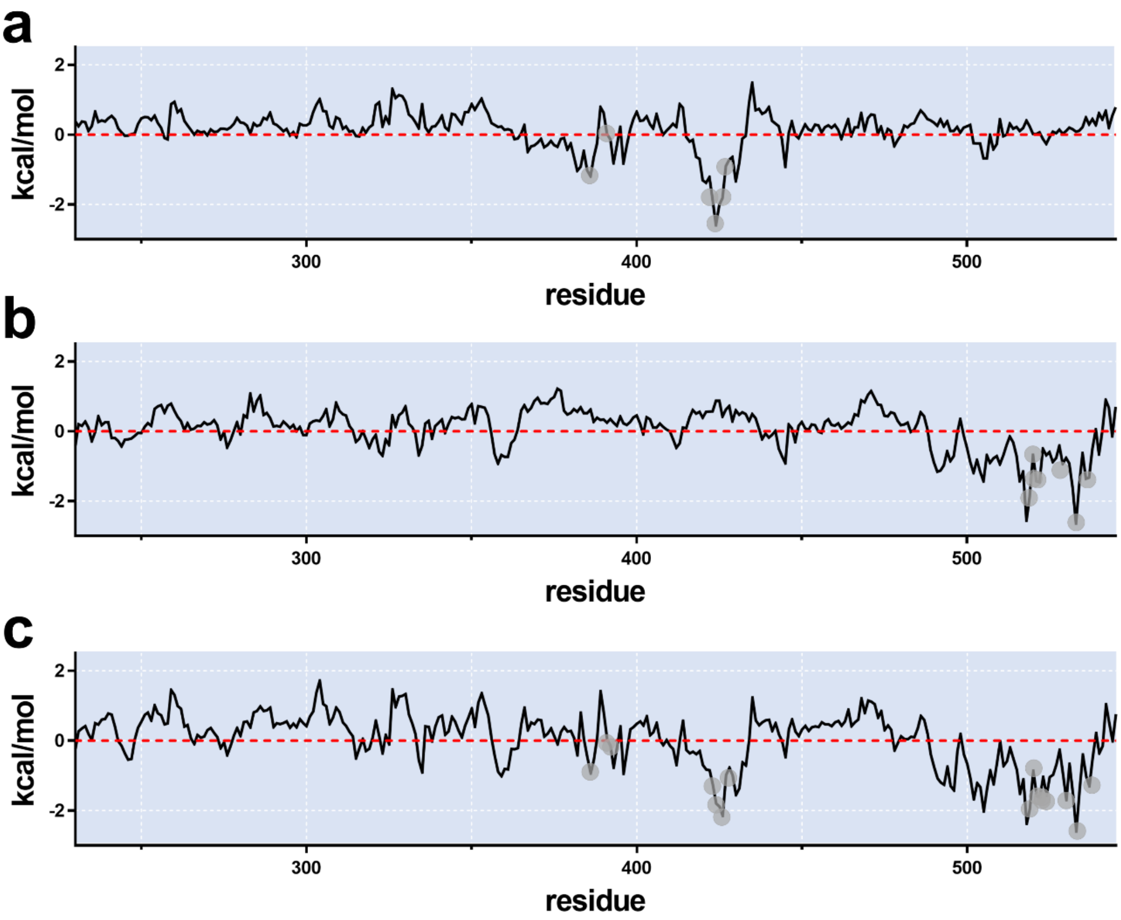

Supplementary Fig. 8 Per-residue allosteric free energy (Δg_i) plots obtained from AlloSigMA analyses for **a** hsEH CTD C423S; **b** hsEH CTD C522S; **c** hsEH CTD WT. Positive and negative Δg_i values correspond to increased and decreased free energies associated to the change in stability due to allosteric signalling. The amino acids of the putative binding sites are highlighted with grey circles.

References

1. Krissinel, E. & Henrick, K. Detection of protein assemblies in crystals. *Comput. Life Sci.* **3695 LNBI,** 163–174 (2005).

2. Steiner, R. A., Lebedev, A. A. & Murshudov, G. N. Fisher's information in maximum-likelihood macromolecular crystallographic refinement. *Acta Crystallogr. - Sect. D Biol. Crystallogr.* **59,** 2114–2124 (2003).

3. Abis, G., Charles, R. L., Eaton, P. & Conte, M. R. Expression, purification, and characterisation of human soluble Epoxide Hydrolase (hsEH) and of its functional C-terminal domain. *Protein Expr. Purif.* **153,** (2019).

4. The PyMOL Molecular Graphics System, Version 1.2r3pre, Schrödinger, LLC.

5. Henikoff, S. & Henikoff, J. G. Amino acid substitution matrices from protein blocks. *Proc. Natl. Acad. Sci. U. S. A.* **89,** 10915–10919 (1992).
