# Supplementary material for: 15-deoxy-Δ12,14-Prostaglandin J2 inhibits human soluble epoxide hydrolase by a dual orthosteric and allosteric mechanism
Source: Commun Biol. 2019 May 17;2:188. doi: 10.1038/s42003-019-0426-2 (PMC6525171; doi:10.1038/s42003-019-0426-2)
Supplement: Supplementary file 2 — Supplementary data [file 42003_2019_426_MOESM2_ESM.pdf]

Supplementary Data

Supplementary Data 1

WT

Time point [min]	Average area	SEM	n
30	41.62841	5.046733	4
150	58.28095	3.673037	4
1200	81.38803	3.077136	4

C522S

Time point [min]	average	SEM	n
30	53.96604	2.34238	3
150	69.88565	3.337473	3
1200	72.31139	3.19296	3

C423S

Time point [min]	average	SEM	n
30	33.28744	4.828815	3
150	35.98099	2.87813	3
1200	64.11715	1.324345	3

Supplementary Data 1 Source data for the 15d-PGJ$_2$ rate of adduction plot reported in Fig. 2e.

Supplementary Data 2

untreated

protein	Activity [$nmol_{6M2N}$ min^{-1}]	SEM	n
WT	0.00782467	0.00057251	6
C423S	0.00928567	0.00093085	6
C522S	0.0101438	0.00044228	5
C423S/C522S	0.00882167	0.00066196	5

Buffer treated elution fractions (apoproteins)

protein	Activity [$nmol_{6M2N}$ min^{-1}]	SEM	n
WT	0.00890555	0.00130759	6
C423S	0.008548	0.00106144	6
C522S	0.008214	0.00111106	5
C423S/C522S	0.00778833	0.00131115	5

15d-PGJ_2 treated flow-through fractions (adducts)

protein	Activity [$nmol_{6M2N}$ min^{-1}]	SEM	n
WT	0.0013999	0.00024933	6
C423S	0.003327	0.00041733	6
C522S	0.0021736	0.00020351	5
C423S/C522S	ND	ND	ND

15d-PGJ_2 treated elution fractions (apoproteins)

protein	Activity [$nmol_{6M2N}$ min^{-1}]	SEM	n
WT	0.008063	0.00079455	6
C423S	0.00775967	0.00130012	6
C522S	0.008747	0.00093452	5
C423S/C522S	0.0097245	0.0004955	5

Supplementary Data 2 Source data for the comparative enzymatic activity analysis reported in Fig. 3c and Supplementary Fig. 3b.
